# A splice-switching oligonucleotide treatment ameliorates glycogen storage disease type 1a in mice with *G6PC* c.648G>T

**DOI:** 10.1172/JCI163464

**Published:** 2023-12-01

**Authors:** Kentaro Ito, Go Tajima, Chikako Kamisato, Miyuki Tsumura, Mitsuhiro Iwamoto, Yukiko Sekiguchi, Yukinobu Numata, Kyoko Watanabe, Yoshiyuki Yabe, Satomi Kanki, Yusuke Fujieda, Koichi Goto, Yoshitaka Sogawa, Masataka Oitate, Hiroyuki Nagase, Shinnosuke Tsuji, Tomohiro Nishizawa, Masayo Kakuta, Takeshi Masuda, Yoshiyuki Onishi, Makoto Koizumi, Hidefumi Nakamura, Satoshi Okada, Masafumi Matsuo, Kiyosumi Takaishi

**Affiliations:** 1 Specialty Medicine Research Laboratories I, Daiichi Sankyo Co., Ltd., Tokyo, Japan.; 2 Department of Pediatrics, Hiroshima University Graduate School of Biomedical and Health Sciences, Hiroshima, Japan.; 3 Division of Neonatal Screening, Research Institute, National Center for Child Health and Development, Tokyo, Japan.; 4 Modality Research Laboratories,; 5 Drug Metabolism and Pharmacokinetics Research Laboratories,; 6 Translational Science Department II, and; 7 Medicinal Safety Research Laboratories, Daiichi Sankyo Co., Ltd., Tokyo, Japan.; 8 Department of Research and Development Supervision, National Center for Child Health and Development, Tokyo, Japan.; 9 Research Center for Locomotion Biology, Kobe Gakuin University, Kobe, Japan.

**Keywords:** Metabolism, Therapeutics, Gene therapy, Genetic diseases, Glucose metabolism

## Abstract

Glycogen storage disease type 1a (GSD1a) is caused by a congenital deficiency of glucose-6-phosphatase-α (G6Pase-α, encoded by *G6PC*), which is primarily associated with life-threatening hypoglycemia. Although strict dietary management substantially improves life expectancy, patients still experience intermittent hypoglycemia and develop hepatic complications. Emerging therapies utilizing new modalities such as adeno-associated virus and mRNA with lipid nanoparticles are under development for GSD1a but potentially require complicated glycemic management throughout life. Here, we present an oligonucleotide-based therapy to produce intact G6Pase-α from a pathogenic human variant, *G6PC* c.648G>T, the most prevalent variant in East Asia causing aberrant splicing of *G6PC*. DS-4108b, a splice-switching oligonucleotide, was designed to correct this aberrant splicing, especially in liver. We generated a mouse strain with homozygous knockin of this variant that well reflected the pathophysiology of patients with GSD1a. DS-4108b recovered hepatic G6Pase activity through splicing correction and prevented hypoglycemia and various hepatic abnormalities in the mice. Moreover, DS-4108b had long-lasting efficacy of more than 12 weeks in mice that received a single dose and had favorable pharmacokinetics and tolerability in mice and monkeys. These findings together indicate that this oligonucleotide-based therapy could provide a sustainable and curative therapeutic option under easy disease management for GSD1a patients with *G6PC* c.648G>T.

## Graphical abstract

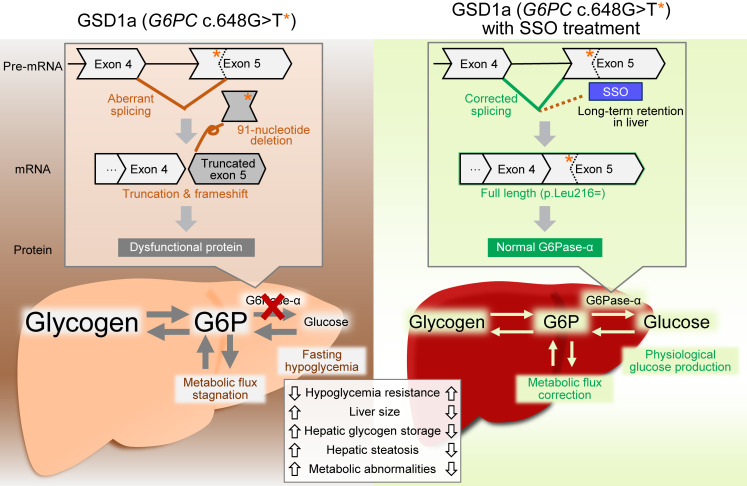

## Introduction

Glycogen storage diseases are rare genetic disorders characterized by the dysfunction of enzymes involved in glycogen metabolism (1). Glycogen storage disease type 1a (GSD1a, OMIM: 232200) is an autosomal recessively inherited inborn error of metabolism defined by impairment of glucose-6-phosphatase-α (G6Pase-α, encoded by the *G6PC* gene) with a prevalence of approximately 1 in 100,000 individuals (2–4). G6Pase-α is the enzyme involved in the hydrolysis of glucose-6-phosphate (G6P) and the production of glucose as the last step of glycogenolysis and gluconeogenesis and is mainly expressed in liver, kidney, and small intestine. Patients with GSD1a manifest severe fasting hypoglycemia due to impaired glucose production and secondary metabolic abnormalities (lactic acidosis, hyperuricemia, hyperlipidemia) associated with excessive G6P production. These metabolic abnormalities lead to the accumulation of glycogen and fat in the liver and kidney, resulting in hepatomegaly and nephromegaly. GSD1a was once a fatal disease in infancy due to severe hypoglycemia, but the life expectancy and prognosis have been substantially improved by introducing frequent (every 4–6 h) intake of a starch-rich diet against life-threatening hypoglycemia as a standard of care. Despite the heavy burden on patients and their families, this approach does not sufficiently prevent biochemical hypoglycemia, glycogen accumulation, or other metabolic abnormalities that lead to hepatocellular adenoma/carcinoma (HCA/HCC) and renal injury (3–8).

The only curative treatment for these patients is liver/kidney transplantation (9), but this is also associated with various difficulties. Thus, curative and less invasive therapies are highly desired. Several gene replacement therapies based on viral vectors have shown promise in correcting hypoglycemia and preventing various complications in GSD1a animal models (10–14), among which an adeno-associated virus–based (AAV-based) product is currently being evaluated in clinical trials (e.g., ClinicalTrials.gov, NCT03517085). However, emerging evidence suggests that AAV therapies may not endure as a lifelong treatment option for patients, and readministration might also be ineffective (15–17). Aside from gene replacement therapies, mRNA replacement therapy based on lipid-nanoparticle technology, which delivers the mRNA to the liver, has also shown beneficial effects on the symptoms in GSD1a model mice (18, 19) and is under evaluation in its first clinical trial (ClinicalTrials.gov, NCT05095727). Although it is likely to be a lifelong treatment option for GSD1a, it may still require complicated dietary management as a result of *G6PC* promoter–independent G6Pase-α levels not being regulated by glucose homeostasis control and a rapid decline in efficacy with a short half-life of the translated protein (19, 20).

To develop a lifelong and easy-to-manage GSD1a treatment, we evaluated an oligonucleotide-based therapy to produce intact G6Pase-α from a prevalent pathogenic human variant, *G6PC* c.648G>T. This variant is particularly identified in East Asian patients with GSD1a, accounting for 91%, 75%, and 54% of the alleles in Japanese, Korean, and Chinese patients, respectively (21). This variant causes aberrant splicing, in which 91 nucleotides from the 5′ end of exon 5 are removed, resulting in the loss of G6Pase-α activity (22, 23). If this aberrant splicing is corrected, the spliced mRNA is translated into intact G6Pase-α protein (p.Leu216=), which would ameliorate disease symptoms. Some antisense oligonucleotides (ASOs) have been investigated as splice-switching oligonucleotides (SSOs) to modulate the targeted splicing and produce functional proteins (24). For GSD1a with *G6PC* c.648G>T, several SSOs with conventional oligonucleotide chemistry achieved the correction of aberrant splicing using immortalized blood cells derived from patients with GSD1a carrying this variant (25, 26). However, to our knowledge, the recovery of G6Pase and the suggested therapeutic effect of SSO treatment have not yet been demonstrated in human cells or in vivo.

A critical limitation of ASO/SSO efficacy in vivo lies in inefficient delivery to pharmacological target tissues (27). Recently, chemical modification and liver-targeting technologies for oligonucleotides have been advanced to resolve these problems. One of the most advanced technologies involves GalNAc-conjugated phosphorothioate ASOs (GalNAc-conjugated PS-ASOs), which are already undergoing clinical trials (28). These oligonucleotides exhibit rapid and extensive liver distribution, long retention, the need for less frequent administration, and a favorable safety profile (29–32), suggesting a therapeutic potential not addressed by other therapies.

Against this background, we designed and synthesized a GalNAc-conjugated PS-ASO, DS-4108b, to achieve a strong and long-term effect on the aberrant splicing in the liver. We also generated *G6PC* c.648G>T conditional knockin (cKI) mice as GSD1a model mice. This work revealed the effect of DS-4108b treatment on the pathophysiology using GSD1a model mice, thus indicating the therapeutic potential of this SSO to surpass existing treatment options.

## Results

### Potential to correct aberrant splicing resulting from G6PC c.648G>T.

**Figure 1:**
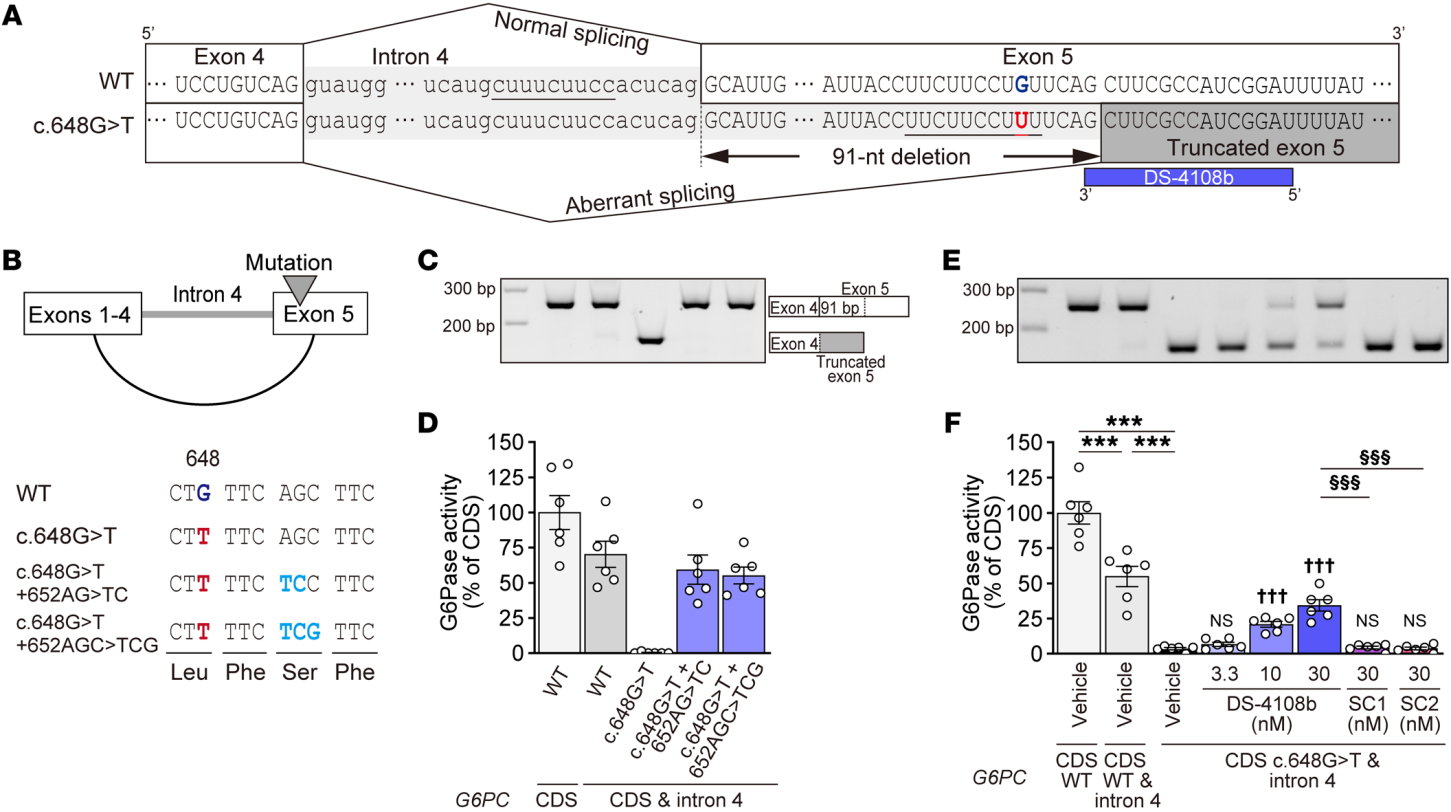
Aberrant splicing caused by *G6PC* c.648G>T and correction by additional mutations or splice-switching oligonucleotide. (**A**) *G6PC* pre-mRNA sequences around the junctions of exon 4–intron 4 and intron 4–exon 5. Sequences with underlining indicate the polypyrimidine tract. The blue bar represents DS-4108b. (**B**) Structure of *G6PC* expression plasmid and sequences around the inserted mutations. Black and dark blue letters indicate nucleotides from the WT *G6PC* sequence, while red and light blue letters represent replaced nucleotides. (**C** and **E**) Agarose gel electrophoresis of RT-PCR products and (**D** and **F**) G6Pase activities of the cell lysates relative to the mean of the WT *G6PC* CDS expression plasmid–transfected group. Quantification data of G6Pase activities are presented as the mean ± SEM (*n* = 6). (**F**) For the vehicle-treated groups, Tukey’s multiple-comparison test was performed (***P < 0.001). For the groups transfected with *G6PC* plasmid containing c.648G>T and intron 4, Dunnett’s multiple-comparison test was performed to compare the DS-4108b– and semiscrambled control ASO–treated groups with the vehicle-treated group (^†††^*P* < 0.001; NS, *P* > 0.05) and the SC1- and SC2-treated groups with the 30 nM DS-4108b–treated group (^§§§^*P* < 0.001).

*G6PC* c.648G>T causes misrecognition of c.652AG as the 3′ end of intron 4 and splicing out of the first 91 nucleotides of WT exon 5 (Figure 1A). To test the hypothesis that correction of this misrecognition restores intact G6Pase-α, we designed *G6PC* expression plasmids containing intron 4, c.648G>T substitution, and additional synonymous substitutions of nucleotides key for this misrecognition (Figure 1B), followed by transfection into 293A cells. Reverse transcription PCR (RT-PCR) analysis revealed that c.648G>T produced aberrantly spliced mRNA, as seen in the patients, whereas the coexistence of c.648G>T and c.652AG>TC or c.652AGC>TGG produced the correctly spliced mRNA (Figure 1C). G6Pase activity for each group relative to that in the *G6PC* coding sequence (CDS) WT plasmid–treated group (100% ± 12%) is shown in Figure 1D. The activity rate of the group treated with the intron 4–inserted plasmid was 70.3% ± 9.3%, indicating a decrease in translation efficiency due to the splicing of intron 4. As expected from the RT-PCR analysis, we found that G6Pase activity was depleted by c.648G>T substitution in the plasmid, while this depletion was almost completely rescued by the additional substitutions. These results support the hypothesis that obstructing the construction of the aberrant splicing acceptor site can correct the aberrant splicing and restore intact G6Pase-α. To apply this finding for therapeutic treatment, we cotransfected DS-4108b, designed to be complementary to the aberrant acceptor site, and *G6PC* expression plasmids with intron 4 and c.648G>T into 293A cells. DS-4108b corrected the aberrant splicing and produced an mRNA variant with 91 nucleotides included in the correct site of the exon 4–5 junction, while semiscrambled control ASOs (SC1 or SC2, 2 or 4 scrambled nucleotides of the DS-4108b sequence, respectively) did not affect the splicing (Figure 1E and Supplemental Figure 1, A–D; supplemental material available online with this article; https://doi.org/10.1172/JCI163464DS1). The splicing corrections were reflected in the dose-dependent recovery of G6Pase activity (Figure 1F). Thus, the SSO DS-4108b can produce intact G6Pase-α from the mutated *G6PC* gene transcript in human cells based on hybridization-dependent inhibition of misrecognition of the 5′ splicing acceptor site.

### Generation and pathophysiological characterization of G6PC c.648G>T–cKI mice.

**Figure 2:**
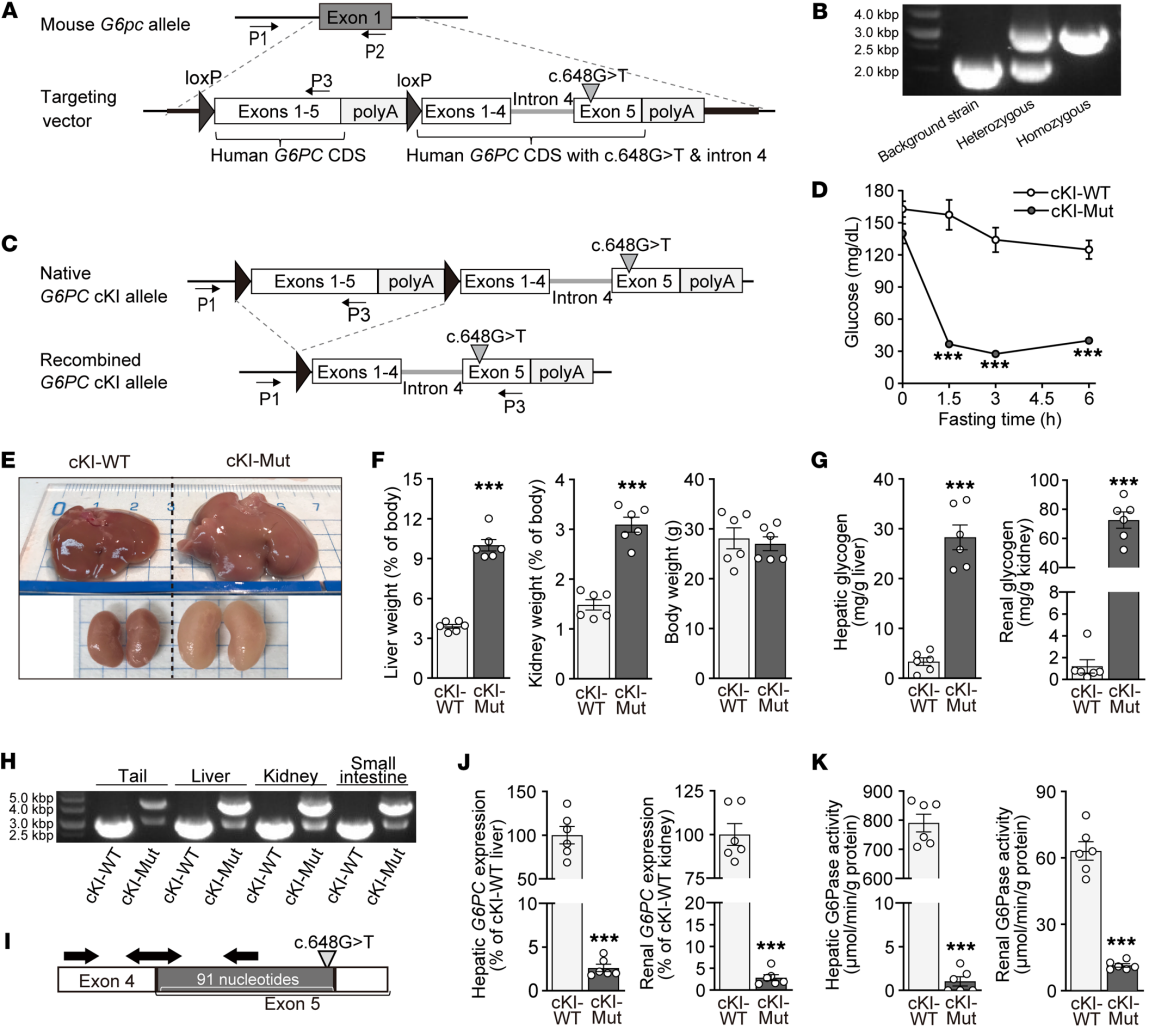
Generation and evaluation of *G6PC* c.648G>T–cKI mice. (**A**) KI scheme of the human *G6PC* gene cassette. Arrows with P1, P2, and P3 indicate the genotyping primers. (**B**) Agarose gel electrophoresis of genotyping PCR of mouse ear DNA with the P1, P2, and P3 primers. Amplicons around 2,000 and 3,000 bp are represented as mouse *G6pc*- and *G6PC*-cKI alleles, respectively. (**C)** Schematic diagrams of Cre/loxP recombination in the *G6PC*-cKI allele. (**D**) Blood glucose levels in a 6-hour fasting test. (**E**) Representative images of whole liver and kidneys. (**F**) Weights of liver, both kidneys, and body. (**G**) Glycogen concentration. (**H**) Agarose gel electrophoresis of genotyping PCR of DNA from tissues with P1 and P3. Amplicons around 3,000 and 4,500 bp are represented as the native and recombined *G6PC*-cKI alleles, respectively. (**I**) Hybridizing sites of specific primers (right and left arrows) and probe (double-headed arrow) for correctly spliced *G6PC* mRNA. (**J**) Relative expression levels of correctly spliced *G6PC* mRNA. (**K**) G6Pase activity. All quantification results are presented as the mean ± SEM (*n* = 6) and were analyzed by unpaired, 2-tailed *t* test to compare cKI-WT and cKI-Mut mice. ***P < 0.001.

**Figure 3:**
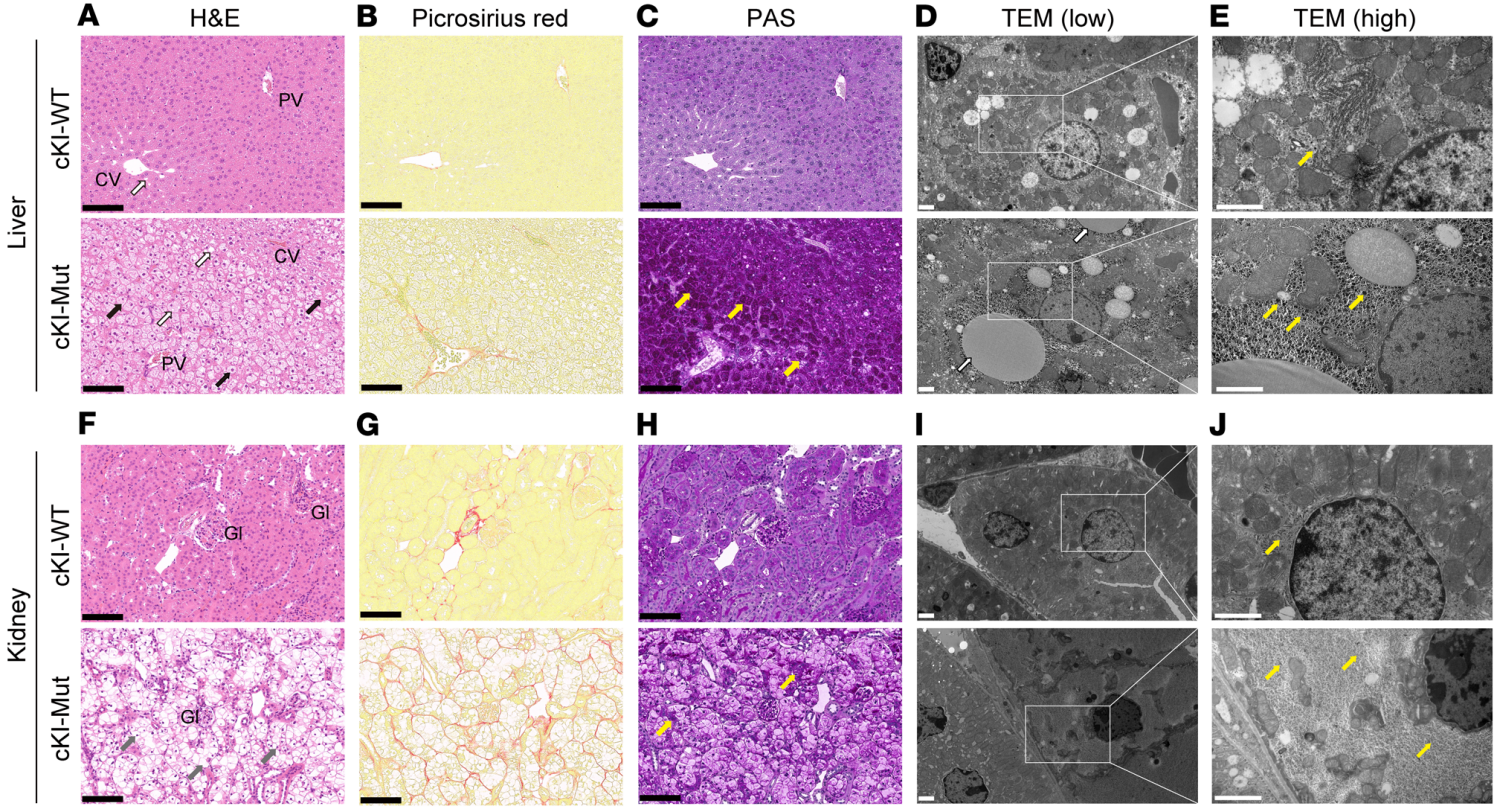
Histopathology of liver and kidney in cKI-WT and cKI-Mut mice. (**A**–**C** and **F**–**H**) Representative stained formalin-fixed, paraffin-embedded (FFPE) sections of liver and kidney from cKI-WT and cKI-Mut mice. H&E-stained liver (**A**) and kidney (**F**). CV, PV, and Gl represent central vein, portal vein, and glomerulus, respectively. White and gray arrows indicate lipid droplets and vacuolar degeneration, respectively. Picrosirius red–stained liver (**B**) and kidney (**G**). Red-stained areas show collagen fibers. PAS-stained liver (**C**) and kidney (**H**). All black and yellow arrows indicate stored glycogen. Black scale bars: 100 μm. (**D**, **E**, **I**, and **J**) Representative TEM images of liver (**D** and **E**) and kidney (**I** and **J**) from cKI-WT and cKI-Mut mice. Black dots indicated by yellow arrows are granules. White scale bars: 2 μm.

To evaluate the therapeutic potential of DS-4108b in vivo, we designed and generated a transgenic mouse strain with human *G6PC*-cKI at the mouse *G6pc* locus, which allows conversion of *G6PC* gene expression by Cre/loxP recombination from the human WT *G6PC* CDS to human *G6PC* c.648G>T with intron 4 (Figure 2, A–C). The *G6PC*-cKI homozygous and Cre-positive mice were subjected to Cre/loxP recombination treatment and defined as cKI mutant (cKI-Mut) mice. The *G6PC*-cKI homozygous and Cre-negative mice without the recombination treatment were defined as cKI-WT mice and used as a normal control. To clarify the pathophysiological characteristics of cKI-WT and cKI-Mut mice, a fasting test and tissue analysis were performed. The blood glucose levels at 6 hours of fasting reached the minimum approximately 2 weeks after completion of the recombination procedure and were maintained at lower levels for the long term (Supplemental Figure 2A). Six weeks after the recombination procedure, cKI-WT and cKI-Mut mice were subjected to a 6-hour fasting test, followed by tissue and plasma sampling. cKI-WT mice maintained their blood glucose levels at around 150 mg/dL. On the other hand, cKI-Mut mice showed a drastic reduction of their blood glucose levels to as low as 30 mg/dL within approximately 3 hours of fasting and maintained these lower levels from 3–6 hours of fasting (Figure 2D). The blood lactate levels in cKI-Mut mice were higher than those in cKI-WT mice under normoglycemic conditions and were not increased but rather decreased under hypoglycemic conditions (Supplemental Figure 2B), as was the case for GSD1a model mice of another strain (33). Tissue analysis also supported the similarity of cKI-Mut mice to tissue from patients with GSD1a. The liver and kidneys of cKI-Mut mice were enlarged and pale (Figure 2E) and accounted for 10.0% ± 0.4% and 3.09% ± 0.15% of total body mass, respectively, whereas those of cKI-WT mice looked normal and accounted for 3.91% ± 0.15% and 1.48% ± 0.11%, respectively (Figure 2F and Supplemental Figure 2A). A much greater amount of glycogen was accumulated in the liver and kidneys of cKI-Mut mice than in those of cKI-WT mice (Figure 2G). As we expected, efficient recombination by the Cre/loxP system was clearly evident in the liver, kidneys, and small intestine (Figure 2H). Using a primer-probe set to specifically detect correctly spliced *G6PC* mRNA (Figure 2I), we observed almost complete depletion of correctly spliced *G6PC* mRNA expression and G6Pase activity in the liver and kidneys of cKI-Mut mice (Figure 2, J and K). H&E staining and Picrosirius Red staining of the liver (Figure 3, A and B) revealed marked hepatic steatosis with enlarged hepatocytes containing lipid and glycogen, especially in the periportal area, although hepatic fibrosis was not apparent. Periodic acid–Schiff (PAS) staining (Figure 3C) and transmission electron microscopy (TEM) (Figure 3, D and E) confirmed the remarkable accumulation of glycogen in liver. We also observed morphological alterations of kidney cortex from cKI-Mut mice. H&E and Picrosirius Red staining revealed enlarged tubular epithelial cells with severe vacuolar degeneration and mild fibrosis on the basolateral side (Figure 3, F and G). The observations of PAS staining and TEM indicated that the degenerated vacuoles consisted of abnormally accumulated glycogen (Figure 3, H–J). In contrast to cKI-Mut mice, we observed no marked histological features in the liver or kidneys of cKI-WT mice. Together, these findings show that cKI-Mut mice successfully mimicked the clinical and biological manifestations of patients with GSD1a and appeared to be a suitable animal model to evaluate the therapeutic potential of human *G6PC* sequence–specific approaches for GSD1a.

### Therapeutic effect of multiple doses of DS-4108b on cKI-Mut mice.

**Figure 4:**
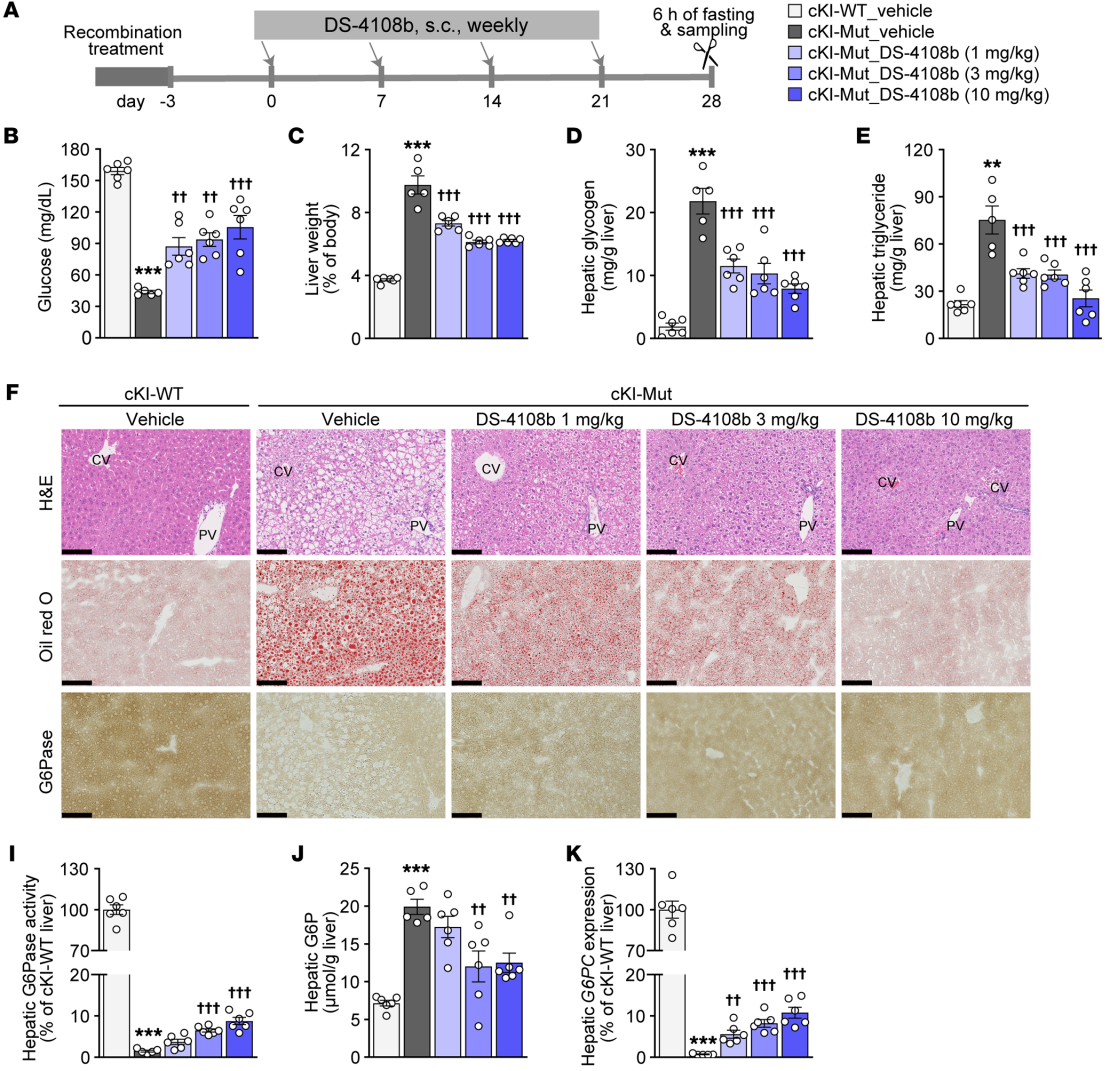
Therapeutic effect of multiple doses of DS-4108b on cKI-Mut mice. (**A**) Experimental design. (**B**) Blood glucose levels after 6 hours of fasting. (**C**) Liver weight relative to BW. (**D** and **E**) Hepatic concentrations of glycogen (**D**) and triglycerides (**E**). (**F**) FFPE liver sections with H&E staining. CV, central vein; PV, portal vein. (**G** and **H**) Fresh-frozen liver sections with Oil Red O staining (**G**) and G6Pase activity staining (brown coloration is proportional to the G6Pase activity) (**H**). Scale bars: 100 μm. (**I**) Hepatic G6Pase activity relative to the mean of the cKI-WT mouse group. (**J**) Hepatic G6P concentration. (**K**) Gene expression of correctly spliced human *G6PC* in liver relative to the mean of the cKI-WT mouse group. For all bar graph panels, quantification results are presented as the mean ± SEM (*n* = 5–6). The vehicle-treated cKI-Mut mouse group was compared with the vehicle-treated cKI-WT mouse group by unpaired, 2-tailed *t* test; *P < 0.01 and ***P < 0.001. DS-4108b–treated cKI-Mut mouse groups were compared with the vehicle-treated cKI-Mut mouse group by Dunnett’s multiple-comparison test; ^††^*P* < 0.01 and ^†††^*P* < 0.001.

To examine the therapeutic potential of DS-4108b to favorably affect the disease manifestations observed in cKI-Mut mice, the mice were assigned to groups on the basis of fasting glucose levels and administered DS-4108b subcutaneously every week for 4 weeks starting 3 days after completion of the recombination treatment. cKI-WT and cKI-Mut mice were fasted for 6 hours and then sacrificed 28 days after the first administration (Figure 4A). The severe hypoglycemia (43.8 ± 1.5 mg/dL) exhibited in cKI-Mut mice at 6 hours of fasting was significantly improved by DS-4108b treatment (Figure 4B). In addition, the increased liver/BW ratio and accumulation of hepatic glycogen and triglycerides in cKI-Mut mice were significantly lowered by the administration of multiple doses of DS-4108b in a dose-dependent manner (Figure 4, C–E, and Supplemental Figure 4A). Histological observations by H&E and Oil Red O staining confirmed the hepatic improvement (Figure 4, F and G). Besides, there were no pathological findings suggestive of safety concerns. Correlating with these improvements, the increased plasma levels of aspartate aminotransferase (AST), alanine aminotransferase (ALT), and triglycerides in cKI-Mut mice were also improved in the DS-4108b–treated groups (Supplemental Figure 3, B–D). Plasma levels of total cholesterol and uric acid in cKI-Mut mice did not show clear exacerbation (Supplemental Figure 3, E and F). Enzymatic histochemical analysis, in which brown staining appeared in proportion to the intensity of G6Pase activity, revealed that G6Pase was distributed in almost all hepatocytes in cKI-WT mice, but was not detected in the vehicle-treated cKI-Mut mice. In the liver of DS-4108b–treated cKI-Mut mice, we observed marked restoration of G6Pase activity in most hepatocytes in a uniform and dose-proportional manner (Figure 4H). Biochemical analysis of the liver lysates confirmed that the depleted hepatic G6Pase activity in cKI-Mut mice was significantly improved by DS-4108b treatment (Figure 4I). At the same time, we also observed a decrease in G6P, the G6Pase substrate (Figure 4J), and an increase in normally spliced *G6PC* by DS-4108b (Figure 4K). DS-4108b led to improvements in blood urea nitrogen (BUN) levels, plasma creatinine concentrations, and renal glycogen concentrations of cKI-Mut mice (Supplemental Figure 3, G–I), but we observed no clear correction of the aberrant splicing in kidney, the nephromegaly, or the abnormal renal histology in cKI-Mut mice (Supplemental Figure 3, J–L). These results suggest that DS-4108b could improve fasting hypoglycemia, hepatomegaly, and major complications in GSD1a caused by *G6PC* c.648G>T through correction of the aberrant splicing and the G6Pase activity, especially in liver.

### Metabolomic effect of multiple doses of DS-4108b on cKI-Mut mice.

**Figure 5:**
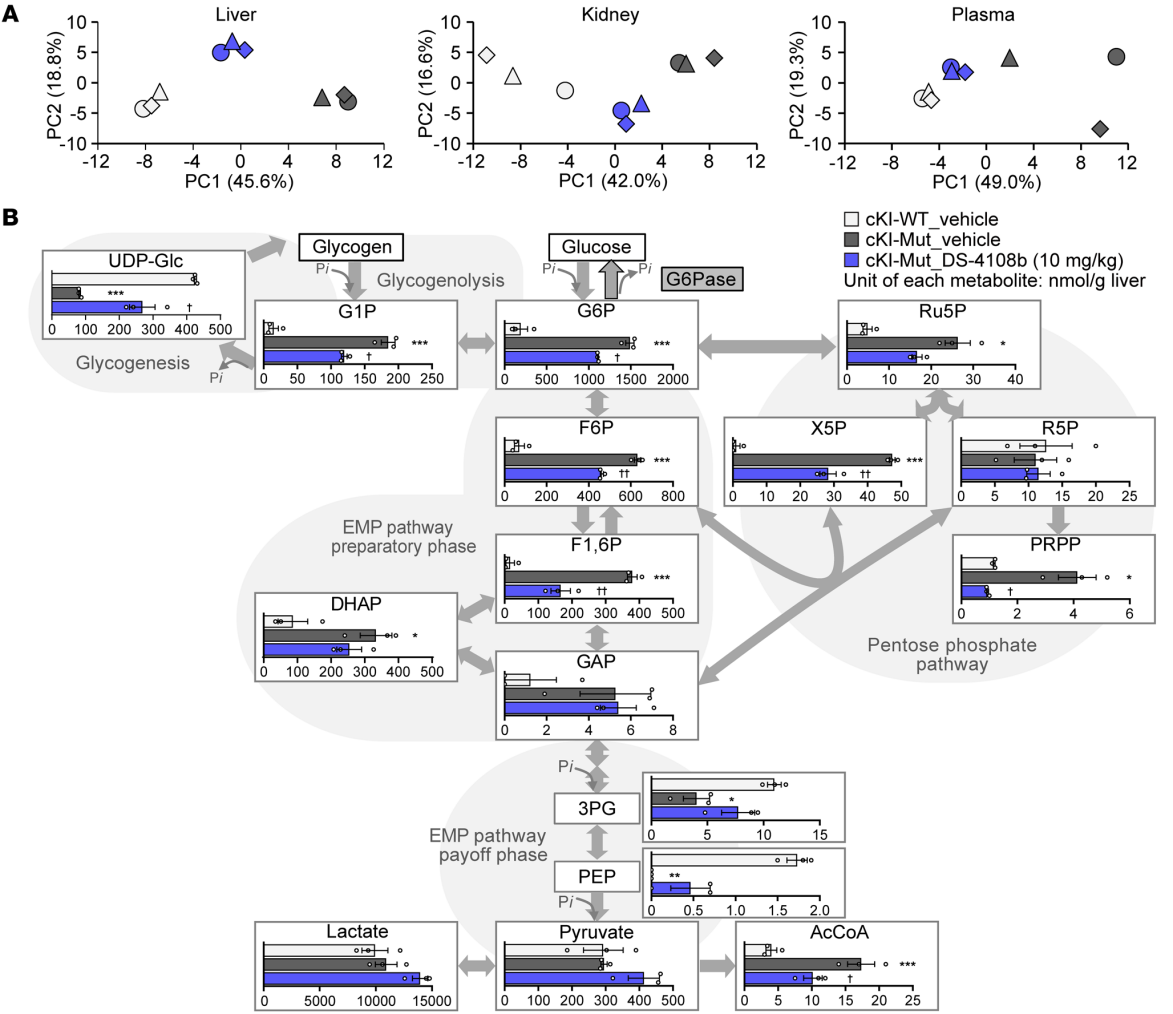
Metabolomics analysis of liver, kidney, and plasma from cKI-Mut mice given multiple doses of DS-4108b. (**A**) The first and second principal components (PC1 and PC2, respectively) of liver, kidney, and plasma in the PCA. Plots from the same individual are presented as the same shape with the same color. Shapes filled with light gray, dark gray, or blue represent groups of vehicle-treated cKI-WT mice, vehicle-treated cKI-Mut mice, and cKI-Mut mice treated with DS-4108b at 10 mg/kg, respectively. (**B**) Metabolic flux changes of key hepatic metabolites for glycogen metabolism, the EMP pathway, and the pentose phosphate pathway. Quantification results are presented as the mean (nmol/g liver) ± SEM (*n* = 3). The vehicle-treated cKI-Mut mouse group was compared with the vehicle-treated cKI-WT mouse group using an unpaired, 2-tailed *t* test. *P < 0.05, **P < 0.01, and ***P < 0.001. DS-4108b–treated cKI-Mut mouse groups were compared with vehicle-treated cKI-Mut mouse group using an unpaired, 2-tailed *t* test. ^†^*P* < 0.05 and ^††^*P* < 0.01. The abbreviations for each metabolite are defined in the Supplemental Methods.

For further clarification of the metabolic changes associated with deficiency and recovery of G6Pase activity in cKI-Mut mice, we performed metabolomics analysis using liver, kidney, and plasma samples from vehicle-treated cKI-WT mice, vehicle-treated cKI-Mut mice, and DS-4108b–treated (10 mg/kg) cKI-Mut mice (Supplemental Tables 1–3). Principal component analyses (PCA) of the metabolites were conducted (Supplemental Tables 4–7) and visualized in 2D scatter plots (Figure 5A) based on the first and second principal components (PC1 and PC2, respectively). PC1 accounted for 45.6%, 42.0%, and 49.0% and PC2 accounted for 18.8%, 16.6%, and 19.3% of the total variation of the metabolites from liver, kidney, and plasma, respectively. Because the PC1 values from DS-4108b–treated cKI-Mut mice were intermediate between those of vehicle-treated cKI-WT mice and vehicle-treated cKI-Mut mice, these results supported the whole-body therapeutic effect of DS-4108b in cKI-Mut mice. We observed clear metabolic changes especially in the liver (Figure 5B). Compared with vehicle-treated cKI-WT liver, there was more accumulation of glucose 1-phosphate and metabolites of the pentose phosphate pathway and the preparatory phase of the Embden-Meyerhof-Parnas (EMP) pathway and less accumulation of uridine diphosphate–glucose (UDP-glucose) and metabolites in the payoff phase of the EMP pathway in vehicle-treated cKI-Mut liver. These changes in hepatic metabolites were attenuated in DS-4108b–treated cKI-Mut mice with recovered G6Pase activity. The elimination of the stagnation of G6P conversion to glucose and inorganic phosphate by DS-4108b clearly reflected the liver metabolic changes. Although DS-4108b did not fully rescue some of the abnormal intermediate metabolite changes due to the G6Pase deficiency of cKI-Mut mice to the same degree as in cKI-WT mice, even at the highest dose, these partial but normalizing trends support the metabolic improvements associated with the recovery of G6Pase activity by DS-4108b. Taken together, these observations support the validity of cKI-Mut mice as animal models of GSD1a pathology and the therapeutic potential of DS-4108b.

### Efficacy onset and duration of single-dose DS-4108b in cKI-Mut mice.

**Figure 6:**
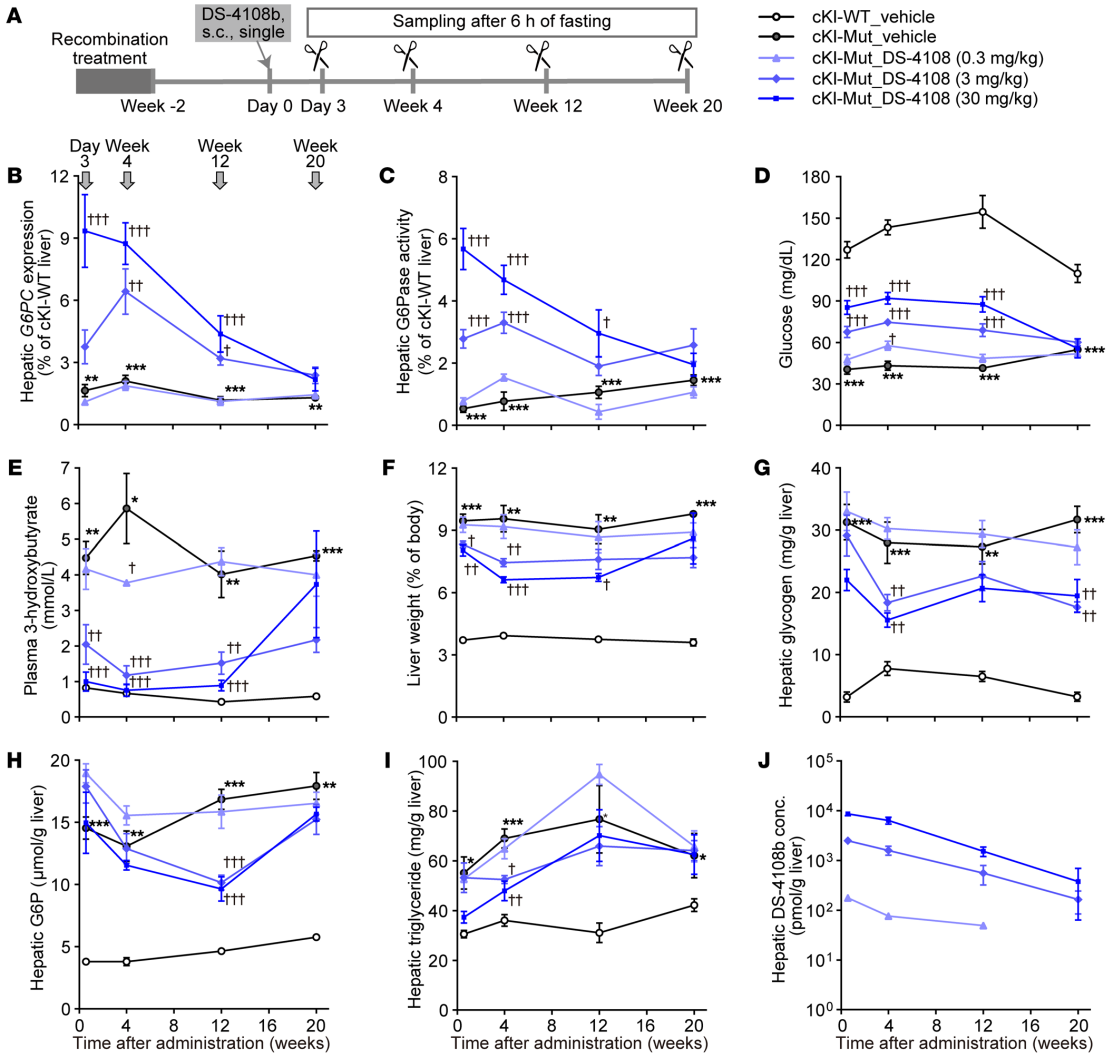
Time-dependent changes of the effect of a single administration of DS-4108b to cKI-Mut mice. (**A**) Experimental design. (**B** and **C**) Gene expression of correctly spliced human *G6PC* (**B**) and hepatic G6Pase activity (**C**) relative to those of the cKI-WT mouse group on the same sampling day. (**D**–**J**) Blood glucose levels (**D**), plasma 3-hydroxybutyrate levels (**E**), liver weight relative to BW (**F**), and hepatic concentrations of glycogen (**G**), G6P (**H**), triglycerides (**I**), and total oligonucleotides originating from DS-4108b, shown as DS-4108b concentration (conc.) (**J**) on the sampling days. For all line graph panels, the quantification results are presented as the mean ± SEM (*n* = 4–5). The vehicle-treated cKI-Mut mouse group was compared with the vehicle-treated cKI-WT mouse group using an unpaired, 2-tailed *t* test at each sampling point. *P < 0.05, **P < 0.01, and ***P < 0.001. DS-4108b–treated cKI-Mut mouse groups were compared with the vehicle-treated cKI-Mut mouse group by Dunnett’s multiple-comparison test at each sampling point. ^†^*P* < 0.05, ^††^*P* < 0.01, and ^†††^*P* < 0.001.

To evaluate the onset and duration of efficacy, DS-4108b was administered subcutaneously to cKI-Mut mice at a dose of 0.3, 3, or 30 mg/kg two weeks after completion of the recombination procedure. Three days, 4 weeks, 12 weeks, and 20 weeks later, cKI-WT and cKI-Mut mice were subjected to a 6-hour fasting test, and then necropsied for the collection of liver, kidney, and plasma (Figure 6A). The correctly spliced *G6PC* mRNA and restored G6Pase activity in liver of cKI-Mut mice increased from day 3, peaked at day 3 or week 4, and then gradually declined (Figure 6, B and C), clearly matching the fasting blood glucose levels (Figure 6D). Plasma levels of 3-hydroxybutyrate, one of the components of ketone bodies and known to increase under hypoglycemic conditions in healthy individuals and even in steady-state GSD1a patients, had almost completely improved in cKI-Mut mice 3 days, 4 weeks, and 12 weeks after administration of 3 or 30 mg/kg DS-4108b (Figure 6E). Correlating with the changes in G6Pase activity in liver and the fasting blood glucose levels, dose-dependent decreases in the liver/BW ratio (Figure 6F and Supplemental Figure 4A), abnormal hepatic accumulation of glycogen (Figure 6G), hepatic G6P (Figure 6H), and hepatic triglycerides (Figure 6I) as well as abnormal plasma triglyceride levels (Supplemental Figure 4B) were observed up to week 12, but diminished at week 20. Histological observations of the liver by H&E staining supported the hepatic biochemical changes (Supplemental Figure 5A). Plasma levels of ALT or uric acid in cKI-Mut mice were not exacerbated (Supplemental Figure 4, C and D). The increased BUN and plasma creatinine concentrations in cKI-Mut mice were attenuated from day 3 in a DS-4108b dose–dependent manner, which lasted more than 4 weeks (Supplemental Figure 4, E and F), but no significant improvements in weight or histological features were observed in cKI-Mut kidney at any time points or at any doses of DS-4108b (Supplemental Figure 4G and Supplemental Figure 5B).

### Pharmacokinetics of DS-4108b following a single administration in mice and monkeys.

**Figure 7:**
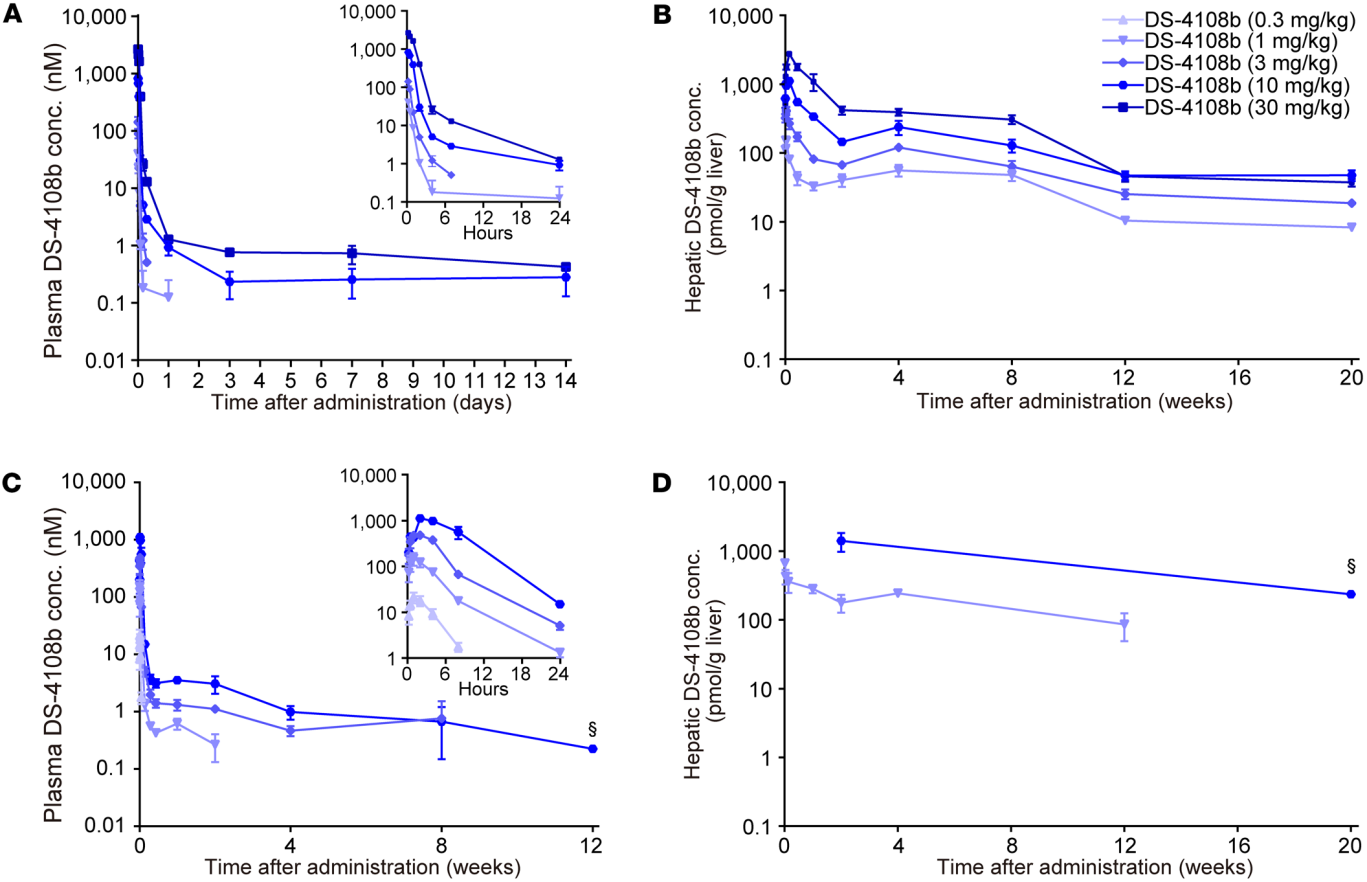
Plasma and liver PK in mice and monkeys following a single administration of DS-4108b. (**A**–**D**) Concentration of total oligonucleotides originating from DS-4108b, shown as DS-4108b concentration, in mouse plasma (**A**), mouse liver (**B**), monkey plasma (**C**), and monkey liver (**D**). Quantification results are presented as the mean ± SEM (*n* = 3–4), except for points marked with § in **C** and **D**, which show the mean (*n* = 2).

In the single-dose efficacy study using cKI-Mut mice, we also assessed the liver pharmacokinetic (PK) profile of DS-4108b. We measured the concentration of total oligonucleotides originating from DS-4108b in the liver using a hybridization-based ligand binding assay, which can detect the PS-ASO moiety of DS-4108b regardless of cleavage of the linker. The measurement revealed that DS-4108b was retained in the liver of cKI-Mut mice for a long period, with a half-life of approximately 4 weeks (Figure 6J), supporting the long-term efficacy described above. For further evaluation of plasma and liver PK, we subcutaneously administered DS-4108b a single time to WT mice (C57BL/6J) and WT monkeys (*Macaca fascicularis*), after which we measured the concentration of total oligonucleotides in plasma and liver. In WT mice, we observed dose-dependent increases in plasma and liver exposure (Figure 7, A and B, and Supplemental Table 8). The total oligonucleotide concentration in plasma decreased rapidly within 1 day, whereas in the liver we observed extensive oligonucleotide distribution. The plasma PK in the post-distribution phase showed a long-lasting profile in parallel to the log scale of the liver PK with a half-life of 4–7 weeks. The liver PK in WT mice was almost the same as that in cKI-Mut mice. Plasma and liver PK in WT monkeys also showed dose-dependent exposure, rapid plasma clearance, and extensive and long-term retention in the liver (Figure 7, C and D). The AUC of the total oligonucleotide concentration in the liver was similar between monkeys at 1 mg/kg and mice at 3–10 mg/kg (Supplemental Table 8).

### Preclinical safety assessments of DS-4108b.

To better ensure safety in clinical trials, DS-4108b was administered subcutaneously to C57B6/J mice at 10, 30, and 100 mg/kg and to cynomolgus monkeys at 3, 10, and 30 mg/kg every 2 weeks for 3 months as a general toxicity study. The effects of DS-4108b on central nervous, cardiovascular, and respiratory systems were also evaluated in the mice and monkeys. There were no apparent safety concerns even at the highest dose in either species (Table 1). To estimate hybridization-dependent off-target effects of DS-4108b on human-specific genes, which could not be evaluated by safety assessment using mice and monkeys, we performed in silico analyses for off-target candidate gene sequences that matched with the DS-4108b complementary sequence and then investigated the homology between them and the corresponding mouse and monkey orthologs (Supplemental Table 9). These analyses did not identify any candidate genes of note for off-target effects that either fully matched the complementary sequence or showed 1 mismatch with it. These results suggest that the preclinical safety profile of DS-4108b makes its acceptable for entry into clinical trials.

## Discussion

The life expectancy of patients with GSD1a has been improved by introducing strict dietary management as the standard of care, but these patients still face life-threatening hypoglycemia, metabolic abnormalities, and the risk of HCA/HCC (3, 4). Therefore, to overcome this serious disease, there is an urgent need for a lifelong curative approach with less complicated disease management.

Here, we demonstrated the promising therapeutic potential of an SSO, DS-4108b, for patients with GSD1a carrying *G6PC* c.648G>T, the most common pathogenic variant in East Asian patients. There were 3 key findings in this study. First, the established GSD1a mouse model closely reflected the pathophysiology of GSD1a resulting from *G6PC* c.648G>T, which can facilitate the prediction of clinical efficacy from preclinical results. Second, DS-4108b recovered G6Pase activity from the *G6PC* c.648G>T gene by correcting the aberrant splicing not only in human cells, but also in the GSD1a model mice with favorable efficacy and PK profiles. This in turn resulted in improvements in hypoglycemia and other complications including liver pathology. Third, DS-4108b had a favorable safety profile in vivo and in silico, supporting its potential as a clinical candidate.

We have shown, for the first time to the best of our knowledge, that SSO restored G6Pase activity from a *G6PC* c.648G>T variant in human cells. Previous SSO studies for *G6PC* c.648G>T demonstrated correction of the aberrant splicing in patient-derived cells (25, 26), but did not show restoration of G6Pase activity, probably due to low *G6PC* expression in the cells. Conventional minigenes often used for SSO evaluation consist only of exons and introns relevant to the target splicing, allowing the evaluation of changes in spliced RNA (34), but not in full-length RNA or at the protein level. Here, we created minigene plasmids containing a full-length *G6PC* CDS with intron 4 to overcome the limitations of conventional minigenes and demonstrated that DS-4108b has potential to correct abnormal splicing by *G6PC* c.648 G>T and restore G6Pase activity in human cells.

Upon further assessment of the therapeutic potential of the SSO treatment, we established a drug-inducible human *G6PC* c.648G>T–KI mouse strain for the following reasons: (a) the preexisting GSD1a animals could be used to assess an SSO acting on specific sequences of human *G6PC* variants because they were established by introducing truncation or mutation into endogenous *G6pc* (33, 35–38). (b) It was expected to be difficult to generate mouse *G6pc* KO with a human *G6PC* c.648G>T–transgenic mouse strain because *G6pc* global KO mice are known to die in weaning due to severe hypoglycemia without frequent glucose supplementation (35). Therefore, we constructed the donor allele by diverting the minigene template and generated a cKI mouse strain that allowed conversion of *G6PC* gene expression in the entire body by Cre/loxP recombination from the human WT *G6PC* CDS to human *G6PC* c.648G>T with intron 4. As expected, the homozygotes with the recombination treatment, i.e., the cKI-Mut mice, showed aberrant splicing and G6Pase deficiency. They also presented a GSD1a-like phenotype, such as fasting hypoglycemia, hepatomegaly, and clear changes in metabolites reflecting the G6Pase-α deficiency.

The cKI-Mut mouse is, to our knowledge, the first GSD1a animal model generated by knocking in a pathogenic human *G6PC* variant, the treatment-related human G6Pase recovery of which could be compared with findings for cKI-WT mice. This direct comparison between human *G6PC* alleles facilitates the translation to a clinical impact in humans by referring to accumulated evidence on the relationship between the GSD1a genotype, phenotype, and G6Pase activity of biopsied liver (23, 39). It should be noted that the effect of DS-4108b on relative G6Pase recovery in cKI-Mut mice may be underestimated by a control comparison with cKI-WT mice. This underestimation is caused by the difference in translation efficiency of *G6PC* without intron 4 in cKI-WT mice and *G6PC* with intron 4 in cKI-Mut mice to G6Pase. Based on the results of minigene assays, the presence of intron 4 may reduce translation efficiency by 50% to 70% (Figure 1, D and F).

As described in Results, DS-4108b showed robust and long-term efficacy with regard to the various abnormalities in cKI-Mut mice, such as fasting hypoglycemia, hepatomegaly, and metabolic changes, suggesting a favorable therapeutic effect in a clinical context. On the other hand, species differences, such as differences in gene sequence and regulation of transcription and metabolism, limit the ability to predict the clinical impact of this preclinical efficacy. To minimize the influence of these limitations and predict reasonable clinical efficacy, we focused on G6Pase activity levels and PK profiles, for which much clinical evidence has been accumulated. The *G6PC* p.P257L variant had approximately 1.2%–6.4% of the G6Pase activity relative to the WT human *G6PC* in cell-based assays (23, 39) and is known as a variant associated with mild symptoms: a carrier homozygous for the variant had never experienced symptomatic hypoglycemia without any dietary management (23). This suggests that if the 10% recovery of hepatic G6Pase activity observed in cKI-Mut in this study is achieved in the target GSD1a patients, it is expected to be sufficient to provide therapeutic benefit for them. Besides, after a single administration, DS-4108b was rapidly taken up and retained in the liver, with a half-life of approximately 4 weeks, which clearly reflected efficacy over a 12-week period for hepatic G6Pase activity, fasting hypoglycemia, and other parameters in cKI-Mut mice. The liver PK in cKI-Mut mice was similar to that in WT mice, suggesting that GSD1a pathophysiology has little effect on the hepatic delivery of DS-4108b. Furthermore, the PK profiles were comparable between monkeys at 1 mg/kg and mice at 3–10 mg/kg and were similar to the findings for other clinically examined GalNAc-ASOs such as pelacarsen (29, 32, 40, 41). It is well known that the PK properties of subcutaneously administered GalNAc-ASOs are comparable between monkeys and humans when normalized on a mg/kg dose basis (29), and the ratio of effective doses in mice to those in humans is approximately 0.15 (42). Given these similarities, subcutaneous administration of DS-4108b to patients with GSD1a is expected to show a PK profile similar to that for monkeys and long-term efficacy similar to that observed in cKI-Mut mice at a lower dose than that administered to WT mice. Thus, DS-4108b is expected to provide sufficient efficacy at a tolerable dose with long dosing intervals of monthly or longer.

This study indicated that DS-4108b restored G6Pase-α from endogenous *G6PC* c.648G>T pre-mRNA, achieved potentially lifelong stable recovery of G6Pase activity with 1 month or longer dosing intervals, and recovered G6Pase activity in most hepatocytes, which are not all achieved by AAV or mRNA therapy. The AAV-transduced *G6PC* gene is considered to be expressed under physiological glycemic control, but only for a portion of the patient’s life (17, 18, 43). In addition, only about 10% of the hepatocytes are expected to be transduced with AAVs, leaving the remaining cells at risk of developing HCA/HCC (11). mRNA therapy could be a chronic GSD1a treatment, but the G6Pase-α level is outside of physiological control and decreases rapidly (*t_1/2_*: 79 h in mice) (19). Hence, mRNA therapy may still require complicated dietary management and weekly hospital visits for intravenous administration. In addition to its efficacy, DS-4108b is considered to have a favorable safety profile as a clinical candidate. No apparent toxicity has been observed in mice or monkeys, even when treated with higher and more frequent doses of DS-4108b than would be used in clinical practice, and there are no fully matched or 1-mismatch/gap genes complementary to DS-4108b except for *G6PC*. The off-target effect on genes with 2 or more mismatches/gap with the ASO-targeting sequence is considered to be very small (44, 45) and is supported by our results showing that the on-target effect of DS-4108b on *G6PC* was also eliminated by the 2-base substitution (SC1, Figure 1, E and F). Taking these findings together, we believe that the efficacy characteristics and safety profile of DS-4108b may fulfill the unmet clinical need.

In this study, there are 2 major limitations, in addition to the species differences mentioned above. One is that the benefit of DS-4108b for renal pathophysiology in clinical practice could not be adequately estimated from the present study. While a tendency toward correction of renal metabolites was observed with weekly DS-4108b administration for 4 weeks, future long-term serial dosing studies are needed to clarify the effect of this favorable tendency on the long-term prognosis for patients with GSD1a. The other limitation is that the disease-inducing conditions and timing of intervention in the established transgenic mice were only partially investigated. Under the current experimental conditions, no hepatic tumorigenicity, renal fibrosis, or glomerular degeneration, as observed in the patients with advanced disease, could be detected for up to 6 months. As differences in pathophysiological severity and therapeutic effects depending on experimental conditions have been reported for other GSD1a mouse models (13, 46), we plan to further examine the effects of the experimental conditions on our transgenic mice. From a different perspective, this study suggests that DS-4108b has favorable efficacy in patients with GSD1a when administered before irreversible tissue remodeling occurs. Fortunately, hepatocellular remodeling such as fibrosis and cirrhosis is rarely observed as a hallmark of patients with GSD1a (3), and DS-4108b could provide clinical benefits for most GSD1a patients with *G6PC* c.648G>T from infancy to adulthood.

In summary, we generated a GSD1a mouse model by homozygous KI of the pathogenic human *G6PC* c.648G>T, the most prevalent variant in East Asia. This model clearly reflected the physiopathology of GSD1a. Using these mice, we obtained in vivo proof of concept, efficacy characteristics, and evidence for the value of translation to a clinical context of the SSO DS-4108b. Although further assessments such as of differences in therapeutic effects due to the timing of intervention are needed, this work highlights the promise of this oligonucleotide-based therapy. This approach has the potential to offer a lifelong, easy-to-manage treatment for patients with GSD1a carrying *G6PC* c.648G>T, who still experience hypoglycemia and develop hepatic complications even under strict and complicated daily dietary management.

## Methods

### Blinding.

Researchers were not blinded to the in vitro experimental conditions or the in vivo treatments of each mouse or monkey.

### ASO preparation.

DS-4108b and semiscrambled control ASOs (SC1, SC2) were synthesized and prepared in the freeze-dried state at Daiichi Sankyo Co., Ltd., as described previously (47). The PS-ASO sequences of DS-4108b, SC1, and SC2 are 5′-AUCCGAUGGCGAAGC-3′, 5′-AUC AGAUGGCGCAGC-3′, and 5′-AUCAGCUGGAGCAGC-3′, respectively (mismatched sequences are underlined). The ASOs were weighed and dissolved in nuclease-free water for cell-based assays. DS-4108b was weighed and dissolved in saline (Otsuka Pharmaceutical Factory) for in vivo assessment. The concentration was calculated from the measured weight, molecular weight, and amount of added solvent, and the purity was not taken into consideration.

### Cell-based assay.

293A cells (Thermo Fisher Scientific) were adherently cultured in maintenance medium, DMEM (high-glucose) with l-glutamine, phenol red, and sodium pyruvate (FUJIFILM Wako Pure Chemical), supplemented with 10% FBS (Thermo Fisher Scientific, lot 1931518) in a CO_2_ incubator (37°C, 5% CO_2_), and were repeatedly passaged using TrypLE Express Enzyme (1′) with no phenol red (Thermo Fisher Scientific). For RNA analysis, 293A cells were plated in a 12-well plate at 2 × 10^5^ cells/1 mL/well and cultured overnight, followed by transfection of plasmid (300 ng/well) and DS-4108b solution using OPTI-MEM I Reduced Serum Medium (Thermo Fisher Scientific) and Lipofectamine 2000 (3 μg/well, Thermo Fisher Scientific). For G6Pase activity analysis, 293A cells were plated in a 6-well plate at 5 × 10^5^ cells/2 mL/well and cultured overnight, and then 2 volumes of transfection solution for RNA analysis were added. Six hours after transfection, the medium with transfection solution was replaced with the maintenance medium. The cells were cultured up to 24 hours after transfection and subjected to respective analyses. The plasmid vectors used in the transfection experiments were generated at Daiichi Sankyo RD Novare Co., Ltd. or Genscript K.K. by insertion of *G6PC* CDS (NM_000151), *G6PC* intron 4 (NC_000017), and mutations into pcDNA3.1. The cell-based assays were conducted as 6 independent trials in singlicate wells.

### Animals.

*G6PC*-cKI mice were generated at the Laboratory Animal Resource Center of the University of Tsukuba. Crl:CD1 (ICR) mice and C57BL/6J mice were purchased from The Jackson Laboratory Japan (formerly Charles River Laboratories Japan). All mating and genotyping to generate homozygous *G6PC*-cKI mice with or without CAGGCre-ER were performed at The Jackson Laboratory Japan. After the transfer of homozygous *G6PC*-cKI mice with or without CAGGCre-ER to Daiichi Sankyo, the mice were housed 3 to 4 per cage during the acclimation period and given rodent chow (FR-2, Funabashi Farm) and tap water ad libitum. C57BL/6J mice for PK analysis were purchased from The Jackson Laboratory Japan and housed under the same conditions as the homozygous *G6PC*-cKI mice. All mice described above were kept under pathogen-free conditions and a 14-hour light/10-hour dark cycle. Monkeys (*Macaca fascicularis*) for PK assessment were purchased from Shin Nippon Biomedical Laboratories, Ltd. and kept under a 14-hour light/10-hour dark cycle at NAS Laboratory, Ltd. or Daiichi Sankyo.

### Generation of G6PC-cKI mice.

We selected 2 guide RNA (gRNA) targets (5′-GGGATCAAGGCCAACCGGGC-3′ and 5′-GTGGTAAGAACCATCCCGAG-3′) located upstream and downstream of exon 1 of *G6pc*, respectively. These 2 sequences were inserted into the pSpgRNA plasmid. We prepared a targeting vector (Figure 2A) with insertion of mouse *G6pc* genome sequences as 5′ and 3′ recombination arms, human *G6PC* CDS, human *G6PC* intron 4, two loxP sequences, and 2 poly-A sequences from bovine growth hormone into the pBluescript II SK (+) plasmid. These 2 pSpgRNA plasmids, the targeting vector, and pSpCas9 carrying a Cas9 expression unit were extracted and purified using the FastGene Plasmid Mini Kit (Nippon Genetics) and filtrated using a MILLEX-GV 0.22 μm Filter unit (Merck Millipore) for microinjection. Pregnant mare serum gonadotropin (5 units) and human chorionic gonadotropin (5 units) were intraperitoneally injected into female C57BL/6J mice at a 48-hour interval, and the mice were mated with male C57BL/6J mice. Zygotes were collected from the oviducts of mated females and microinjected with approximately 2 pL of a mixture of 2 pSpgRNA vectors (5 ng/μL, each), pSpCas9 (5 ng/μL), and targeting vector (10 ng/μL). Subsequently, the surviving zygotes were transferred into the oviducts of pseudopregnant ICR females, and newborns were obtained. Then, mice with the intended KI mutant allele but without random integration were selected as founders (F_0_). The F_0_ mice were transferred to The Jackson Laboratory Japan and mated with C57BL/6J mice. An acquired founder with the intended KI mutant allele was selected, followed by backcrossing with C57BL/6J mice 2 times. The *G6PC*-cKI mice were mated with CAGGCre-ER mice (stock number 004453, The Jackson Laboratory), which were recovered in the same facility. Subsequently, mating between the offspring was repeated to generate homozygous *G6PC*-cKI mice with or without CAGGCre-ER. After genotyping, these mice were transferred to Daiichi Sankyo. After recombination treatment, a nonrepellent state was set as the endpoint. Daily observations were performed in principle, and individual mice that reached the endpoint were euthanized. For mice that died during the evaluation period, only samples that could be collected under the same conditions as for other surviving mice were evaluated.

### Recombination treatment.

Tamoxifen-sunflower seed oil solutions (MilliporeSigma) were prepared at 8 mg/mL and administered intraperitoneally at 10 mL/kg/day for 5 consecutive days to 8- to 10-week-old homozygous *G6PC*-cKI mice with CAGGCre-ER. Two to 3 weeks after the administration, NLS-Cre mRNA (5MeC, Psi) (TriLink BioTechnologies) encapsulated in lipid nanoparticles by NanoAssemblr (Precision Nanosystems) was administered intravenously into the tail at 1 mg/10 mL/kg. The lipid nanoparticles used in this study were particles of an ionizable lipid, phospholipid, cholesterol, and a PEGylated lipid, as previously described (48). The *G6PC*-cKI homozygous and CAGGCre-ER–positive mice with the recombination procedure were defined as cKI-Mut mice. The *G6PC*-cKI homozygous and CAGGCre-ER–negative mice without the recombination procedure were defined as cKI-WT mice.

### Fasting test.

The fasting test was performed during the light period. The mice were first moved into fasting cages (wire net floor, tap water ad libitum, no chow), and the time point at which the fasting started was set as 0 hours. Blood was taken from the tail vein, and glucose levels (mg/dL) were measured with a glucose measuring device (Accu-Chek Aviva and Accu-Chek Aviva Strip F, Roche DC Japan K.K.) after 0, 1.5, 3, and 6 hours of fasting. In some mice, blood lactate levels at each fasting time point were measured with Lactate Pro 2 and the Lactate Pro 2 Sensor (Arkray Marketing). After 6 hours of fasting, some mice were sacrificed for tissue and blood sampling, while others were housed in a normal cage with some blocks of rodent chow spread on the floor.

### Administration of DS-4108b.

cKI-Mut mice were evenly allocated to each group using SAS System Release 9.2 (SAS Institute) based on blood glucose levels at 6 hours of fasting, so that the severity of fasting hypoglycemia would be uniform among the groups in each test (multiple-administrations test and single-administration test) lot. The cKI-WT mice for the efficacy study and the WT mice and monkeys for the PK and safety assessments were randomly assigned to the groups. Just before administration, the animals were weighed, after which concentration-adjusted DS-4108b solution or saline (Otsuka Pharmaceutical Factory) as a vehicle was administered subcutaneously to the mice at a volume of 10 mL/kg. Ages at DS-4108b treatment and endpoints were determined prospectively on the basis of the time course and individual variations of fasting hypoglycemia in cKI-Mut mice.

### Sampling.

cKI-WT and cKI-Mut mice were weighed after measuring blood glucose following 3 hours of fasting. After completing the fasting test, blood was drawn from the inferior vena cava with Heparin Na (Mochida Pharmaceutical Co., Ltd.), and the liver and kidneys were removed from mice under 3% isoflurane anesthesia. Blood was centrifuged (2,000*g*, 5 min, 4°C) to prepare the plasma. The liver was excised, weighed, and then subjected to fixation with 10% neutral buffered formalin, fixation with 2.5% glutaraldehyde in 0.1 M phosphate buffer (pH 7.4), or to unfixed frozen embedment with SCEM (Section-Lab), and stored at room temperature, at 4°C or –80°C, respectively. The rest of the liver was divided into approximately 3 mm cubes and immediately frozen with liquid nitrogen or dry ice. Both kidneys were weighed after removing Gerota’s fascia. One kidney was divided horizontally and subjected to formalin fixation and creation of a fresh-frozen block, as for liver. The other kidney was divided into 4 blocks horizontally and vertically and immediately frozen with liquid nitrogen. The frozen plasma and tissues were stored at –80°C. Male mice and monkeys for PK analysis were subjected to plasma and liver sampling without fasting treatment. Blood was collected intravenously over time or at a certain point and centrifuged (2,000*g*, 5 min, 4°C) to prepare the plasma. After opening the abdomen and bleeding the animals under anesthesia, the required amount of liver was excised and immediately frozen with liquid nitrogen or dry ice.

### Statistics.

Data represent the mean ± SEM and were analyzed with GraphPad Prism, version 9.0 (GraphPad Software) or Microsoft Excel 2013. All statistical analyses were performed using SAS System, Release 9.2, or Microsoft Excel 2013. A *P* value less than 0.05 was considered statistically significant. The statistical analysis method and replicate numbers for each of the in vitro and in vivo assays except the metabolome analysis were determined by a preliminary study. The comparison of each liver metabolite was performed as a post hoc analysis. For statistical comparisons of the G6Pase activity between the transfected 293A cells shown in Figure 1F in the vehicle-treated groups, a 1-way ANOVA with Tukey’s multiple-comparison test was performed, and for the *G6PC* plasmid containing c.648G>T and the intron 4–transfected groups, Dunnett’s multiple-comparison test was performed to compare the means of the DS-4108b–, SC1-, and SC2-treated groups with the means of the vehicle-treated group. For the groups treated with 30 nM ASO, Dunnett’s multiple-comparison test was performed on the means of the SC1- and SC2-treated groups compared with the mean of the DS-4108b–treated group. To evaluate the pathophysiology of cKI-Mut mice, the statistical significance of the differences of the vehicle-treated or untreated cKI-Mut mouse group from the cKI-Mut mouse group was analyzed by unpaired, 2-tailed *t* test. To evaluate the in vivo efficacy of DS-4108b on the pathophysiology of cKI-Mut mice at each time point, the DS-4108b–treated cKI-Mut mouse groups were compared with the vehicle-treated cKI-Mut mouse group using a parametric Dunnett’s multiple-comparison test or an unpaired, 2-tailed *t* test. Data in the figures represent the mean ± SEM.

### Study approval.

All animal experimental procedures were performed with the approval of the animal experimental committee of the Laboratory Animal Resource Center of the University of Tsukuba (Tsukuba, Japan), The Jackson Laboratory Japan(Ishioka, Japan), NAS Laboratory Ltd., (Narita, Japan), Shin Nippon Biomedical Laboratories Ltd., (Kagoshima, Japan), and Daiichi Sankyo Co., Ltd. (Tokyo, Japan).

### Data availability.

All of the data and methods are presented in the manuscript or in the Supplemental Materials. All individual values for figures and tables are shown in the Supplemental Supporting Data Values file.

## Author contributions

KI, GT, ST, M. Koizumi, MM, and KT conceptualized the study. KI, KW, MT, YF, KG, Y Sogawa, H Nagase, ST, and MM designed the study methodology. KI, CK, MI, Y Sekiguchi, YN, KW, YY, SK, YF, KG, M Kakuta, TM, YO, and M Koizumi conducted experiments. KI and CK performed visualization work. KI, MI, Y Sogawa, MO, M Koizumi, and KT were responsible for project administration. GT, H Nakamura, ST, TN, H Nagase, SO, MM, and KT supervised the study. KI wrote the original draft of the manuscript. GT, H Nagase, TN, Y Sogawa, MO, M Koizumi, SO, MM, and KT reviewed and edited the manuscript. All authors read and approved the final manuscript.

## Supplementary Material

Supplemental data

Supplemental table 1

Supplemental table 2

Supplemental table 3

Supplemental table 4

Supplemental table 5

Supplemental table 6

Supplemental table 7

Supplemental table 8

Supplemental table 9

Supporting data values

## Figures and Tables

**Table 1:**
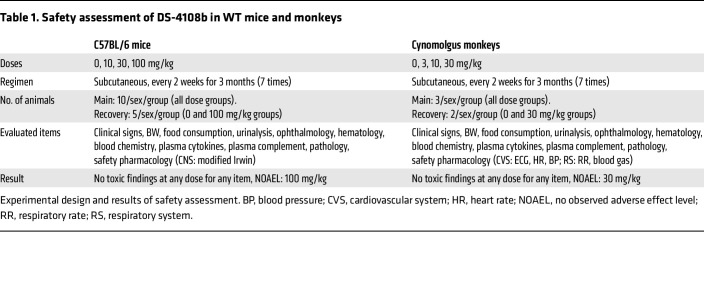
Safety assessment of DS-4108b in WT mice and monkeys

## Version 1 10/03/2023

In-Press Preview

## Version 2 12/01/2023

Electronic publication

## Version 3 12/06/2023

Patent information corrected in ref. 48. Supplemental refs. 49-52 are available in Supplemental Methods.
