# Supplementary material for: A splice-switching oligonucleotide treatment ameliorates glycogen storage disease type 1a in mice with G6PC c.648G>T
Source: J Clin Invest. 2023 Dec 1;133(23):e163464. doi: 10.1172/JCI163464 (PMC10688987; doi:10.1172/JCI163464)
Supplement: Supplemental data [file jci-133-163464-s009.pdf]

Supplemental materials for

A splice-switching oligonucleotide treatment ameliorates glycogen storage disease type 1a in mice with *G6PC* c.648G>T

Authors:

Kentaro Ito, Go Tajima, Chikako Kamisato, Miyuki Tsumura, Mitsuhiro Iwamoto, Yukiko Sekiguchi, Yukinobu Numata, Kyoko Watanabe, Yoshiyuki Yabe, Satomi Kanki, Yusuke Fujieda, Koichi Goto, Yoshitaka Sogawa, Masataka Oitate, Hiroyuki Nagase, Shinnosuke Tsuji, Tomohiro Nishizawa, Masayo Kakuta, Takeshi Masuda, Yoshiyuki Onishi, Makoto Koizumi, Hidefumi Nakamura, Satoshi Okada, Masafumi Matsuo, Kiyosumi Takaishi*

* Corresponding author

This file includes:

Supplemental methods; Supplemental references 49–52; Supplemental Figures 1–5

Other Supplemental materials for this manuscript includes the following:

Supplemental Tables 1 to 9 (Excel files)

Supporting data values (Excel file)

Supplemental methods

Genotyping PCR. Genomic DNA was extracted using DNeasy 96 Blood & Tissue Kit (QIAGEN) in accordance with the kit protocol. To confirm *G6PC* cKI allele numbers and Cre/loxP recombination, multiplex PCR (20 µL per tube; 98℃ for 5 min, 32 cycles of 98℃ for 15 s and 68℃ for 4.5 min, followed by 68℃ for 5 min, and then holding at 12℃) was performed with KOD FX DNA polymerase (TOYOBO) and three primers at 6 pmol each as follows: forward (P1): 5′-TTC CTT CCA AAG CAG GGA CTC TCT ATG T-3′, reverse_1 (P2): 5′-CTT GCA GAA GGA CAA GAC GTA GAA GAC C-3,′ reverse_2 (P3): 5′-AAT GTT CAT TCC TTC CTC CAT CCT TCC T-3′. PCR primers for genotyping were purchased from Eurofins. The PCR product was subjected to gel electrophoresis analysis with 1.5% agarose gel and the genotype or recombination was confirmed. The amplicon sizes of mouse intact allele, native *G6PC* cKI allele, and Cre/loxP recombined allele were estimated at 2246, 3243, and 4739 bp, respectively. To judge CAGGCre-ER^{TM} as positive or negative, Jax-recommended multiplex PCR (25 µL per tube; 95℃ for 2 min, 32 cycles of 95℃ for 30 s, 60℃ for 30 s, and 72℃ for 1 min, followed by 72℃ for 5 min, and then holding at 12℃) was performed with Taq DNA polymerase (Promega) and four primers at 10 pmol each as follows: 5′-GCG GTC TGG CAG TAA AAA CTA TC-3′, 5′-GTG AAA CAG CAT TGC TGT CAC TT-3′, 5′-CTA GGC CAC AGA ATT GAA AGA TCT-3′, and 5′-GTA GGT GGA AAT TCT AGC ATC ATC C-3′. The PCR product was subjected to gel electrophoresis analysis with 1.5% agarose gel and the genotype was confirmed. The amplicon sizes of mouse intact allele and CAGGCre-ER^{TM} allele were estimated as 324 and 100 bp, respectively.

Total RNA extraction and reverse transcription. The cells were lysed using 400 µL/well of RLT buffer in RNeasy Mini QIAcube Kit (QIAGEN) after washing with phosphate-buffered saline (PBS;

FUJIFILM Wako Pure Chemical) and 350 µL of the lysate was dispensed into a 2 mL tube. The tissue block was homogenized with beads and 800 µL of RLT or RLT Plus (QIAGEN) using TissueLyser (QIAGEN), followed by centrifugation (12,000 × g, 3 min, room temperature) and then 350 µL of the lysate supernatant was dispensed into a 2 mL tube. The lysate-containing tube was set in QIAcube (QIAGEN) and the total RNA of the lysate was extracted automatically using RNeasy Mini QIAcube Kit and RNase-Free DNase Set (QIAGEN) based on the QIAcube installed protocol, RNeasy Mini-Animal tissues, and cell-DNase digest. The concentration of the extracted RNA was measured using DropSense16 (Unchained Labs) and 0.4 µg of RNA was subjected to reverse-transcription reaction using High-Capacity RNA-to-cDNA Kit (20 µL per tube; 37°C for 60 min, 95°C for 5 min, and holding at 4°C). After reverse transcription, cDNA solution was diluted 5-fold with nuclease-free water and subjected to RT-PCR and qRT-PCR as a template.

RT-PCR and fragment analysis. Two microliters of the diluted cDNA solution was subjected to PCR reactions (25 µL per tube; 94°C for 5 min, 38 cycles of 98°C for 30 s, 60°C for 30 s, and 68°C for 30 s, then 68°C for 2 min, and finally holding at 4°C) using a Veriti thermal cycler (Thermo Fisher Scientific) with Platinum™ PCR SuperMix High Fidelity Kit (Thermo Fisher Scientific) and the following PCR primers (10 pmol each): forward: 5′-TTGTGGTTGGGATTCTGGGC-3′, reverse: 5′-TCCAGAGTCCACAGGAGGTC-3′. After 5-fold dilution of the PCR-reaction product with nuclease-free water, 20 µL was applied to a well of E-gel EX 2% Agarose (Thermo Fisher Scientific) on the E-Gel iBase™ Power System (Thermo Fisher Scientific). Then, the power system was run for 10 min and a gel image was taken on a ChemiDoc XRS+ system (Bio-Rad Laboratories).The gel slice containing the detected band was excised and subjected to DNA purification using NucleoSpin Gel and PCR Clean-up Kit (Takara Bio). The concentration of purified DNA solution was measured using DropSense16. Eight microliter solutions for sequence analysis were prepared by mixing the purified

DNA (8 ng) and sequence primer (4 pmol, 5′-GCTGTGCAGCTGAATGTCTG-3′) and subjected to sequencing outsourced to Eurofins Genomics using 3730xl DNA Analyzer (Thermo Fisher Scientific). Wave images of the sequence data were obtained by FinchTV version 1.4.0 (Digital World Biology) and alignment diagrams were generated using GENETYX version 13 (GENETYX).

qRT-PCR. Two microliters of the cDNA solution was subjected to quantitative real-time PCR assessment (384-well plate, 10 µL per well, TaqMan, Fast mode, Comparative CT) with QuantStudio 7 Flex (Thermo Fisher Scientific) using TaqMan Fast Advanced Master Mix (Thermo Fisher Scientific) and the following qRT-PCR PCR primers (Thermo Fisher Scientific): correctly spliced *G6PC*: forward: 5′-GCT GCT CAT TTT CCT CAT CAA GTT-3′ (10 pmol), reverse: 5′-TGT GGA TGT GGC TGA AAG TTT CT-3′ (10 pmol), probe 5′-TCC TGT CAG GCA TTG C-3′ (2.5 pmol), housekeeping control gene: mouse Apob (for liver), Mm01545150_m1; mouse Actb (for kidney), and Mm02619580_g1. Using the relative quantification (RQ) value of correctly spliced human *G6PC* to mouse Apob or mouse Actb, which was calculated by the comparative CT method in the instrument program, the *G6PC* gene expression (%) relative to the mean of the vehicle-treated cKI-WT mouse group was calculated.

G6Pase activity. Isotonic buffer was prepared by mixing 0.25 M sucrose, 20 mM HEPES, 1 mM EDTA buffer, and a 1/100 volume of Protease Inhibitor Cocktail (Nacalai Tesque). Cells on a six-well plate were washed with PBS and then 500 µL of the ice-cold isotonic buffer was added to a well. The cell suspension was collected into beads containing crushed tube (Sarstedt) using a cell scraper and pipette and homogenized (speed 6.0, 20 s, 2 times) using Fastprep24 (MP Bio Japan). The tissue block was transferred into beads containing a crushing tube and homogenized (speed 6.0, 20 s, 2 times) with

800 μL of ice-cold isotonic buffer using Fastprep24. After centrifugation (1000*g*, 10 min, 4℃) of the homogenate, the supernatant was collected and centrifuged again (13,000*g*, 60 min, 4℃). Homogenization was conducted with dry ice powder. After removing the supernatant, the remaining pellet was suspended in 50 μL of isotonic buffer and subjected to the measurement of total protein concentration using DC protein assay kit (Bio-Rad Laboratories) with the plate reader SPECTRAmax M4 (Molecular Devices). The pellet suspension was diluted to 1 mg/mL with isotonic buffer, which was named the fraction solution. Then, the inorganic phosphate concentration after the reaction of G6Pase in the fraction solution was measured by the following method: A 3:1 mixture of 100 mM Bis-Tris Buffer, pH 6.5 (Nacalai Tesque), and prepared solution of 200 mM Glucose 6-Phosphate (Sigma-Aldrich) was prepared as G6Pase reaction buffer and dispensed at 200 μL into the well of a 96-well plate. After incubation (5 min, 37°C) for prewarming, 5 μL of the fraction solution was added to the well and incubated (30 min, 37°C) for the G6Pase reaction; then, the reaction was stopped by adding 45 μL of 20% trichloroacetic acid solution (Sigma-Aldrich) and incubating for 5 min at room temperature. The G6Pase-reacted solution was centrifuged (4000*g*, 10 min, room temperature) and the supernatant was subjected to measurement of inorganic phosphate concentration using Taussky-Shorr Color Reagent (Nacalai Tesque) and Phosphorus Standard Solution (FUJIFILM Wako Pure Chemical) by the Taussky-Shorr method. The absorbance (660 nm) was measured using the plate reader SPECTRAmax M4. In parallel with this, the background inorganic phosphate concentration of the fraction solutions was measured as follows: The fraction solution and 20% trichloroacetic acid solution were mixed at 1:9 and incubated for 5 min at room temperature to deactivate proteins; then, 50 μL of the mixture was added to 200 μL of the prewarmed (5 min, 37°C) G6Pase reaction buffer, followed by incubation for 30 min at 37°C. The solutions for background measurement were centrifuged (4000*g*, 10 min, room temperature) and the supernatant was subjected to the measurement of inorganic phosphate concentration as described above. The amount of dephosphorylated inorganic

phosphate (µmol) per unit time (min) of the unit total protein (g) in the fraction was calculated as G6Pase activity (µmol/min/g protein). In the cell-based assay, the G6Pase activity (%) relative to that of *G6PC* CDS plasmid-transfected cells was calculated.

Biochemical analysis.

Blood chemistry: Biochemical parameters in plasma were measured using the following kits: triglyceride: Triglyceride E-test Wako, AST and ALT: Transaminase CII Test Wako, total cholesterol: Cholesterol E-test Wako, uric acid: L-type Wako UA-M, creatinine: L-type Wako CRE-M (FUJIFILM Wako Pure Chemical), BUN: N-assay BUN-L Nittobo D-type R1, R2, and Multichem S (Nittobo Medical), and 3-hydroxybutyrate: EnzyChrom Ketone Body Assay Kit (BioAssay Systems). All levels of absorbance and fluorescence were measured using the plate reader SPECTRAmax M4.

Glycogen: The frozen tissue was weighed and transferred into a 2 mL tube, followed by homogenization with beads and an optimal volume of purified water to make 50 mg/mL using TissueLyser. These procedures were performed under ice cooling. The homogenates were heated (100°C, 10 min) followed by centrifugation (18,000*g*, 10 min, 4°C), after which the glycogen levels in the resulting supernatants were measured using Glycogen Colorimetric/Fluorometric Assay Kit (BioVision). Assays were performed in accordance with the colorimetric method and hepatic glycogen concentration (mg/g liver) was calculated.

Liver triglyceride: The frozen tissue was weighed and transferred into a 2 mL tube, followed by homogenization with beads and 0.5 mL of triglyceride extracting solution (1000:1 mixture of isopropanol and acetic acid using TissueLyser). These procedures were performed under ice cooling. The homogenate was transferred to a 15 mL tube and mixed with 2 mL of a 3:1 mixture of hexane and saline using a rotary mixer for 1 h at room temperature, followed by centrifugation (2000*g*, 5 min,

room temperature). The upper layer was transferred into glass tubes and incubated (40°C, overnight) with a heat block in the fume hood to volatilize the organic solvent. The optimal volume of isopropanol was added into the glass tube to prepare 10 mg tissue/mL solution, followed by measurement of the hepatic triglyceride concentration (mg/g liver) with Triglyceride E-test Wako (FUJIFILM Wako Pure Chemical).

G6P: The frozen tissue was weighed and transferred into a 2 mL tube followed by homogenization with beads and an optimal volume of G6P Assay Buffer contained in the PicoProbe Glucose-6-Phosphate Fluorometric Assay Kit (BioVision) to prepare 100 mg tissue/mL solution using TissueLyser. These procedures were performed under ice cooling. The homogenate was centrifuged (10,000*g*, 10 min, 4 °C) and the supernatant was added to an ultrafiltration spin column 10K (Aproscience) for deproteinization, followed by centrifugation (14,000*g*, 30 min, 4 °C). The concentration of G6P in the deproteinized solution was measured in accordance with the protocol of the kit and used to calculate hepatic G6P concentration (μmol/g liver).

Histopathological analysis. All sectioning, staining, and image capturing were performed at Daiichi Sankyo RD Novare. Formalin-fixed tissues were embedded in paraffin blocks, which were subsequently sectioned into slices that were in turn subjected to H&E, picrosirius red, and PAS staining in accordance with conventional methods. The following key reagents were used for each staining: H&E: Tissuetech Hematoxylin 3G (Sakura Finetech) and Tissuetech Eosin (Sakura Finetech). picrosirius red: Van Gieson Solution A (Muto Pure Chemicals) and Sirius Red C.I. 35780 (FUJIFILM Wako Pure Chemical), and PAS: Cold Schiff’s Reagent (Muto Pure Chemicals), orthoperiodic acid, and Mayer’s Hematoxylin Solution (Muto Pure Chemicals). Sections of SCEM fresh frozen block were prepared and subjected to oil red O staining and staining of G6Pase activity. For oil red O staining, Oil Red O Stain Kit (Sigma-Aldrich) and Mayer’s Hematoxylin Solution were

used. Staining to indicate G6Pase activity was performed as follows: The sections were incubated for 10 min in G6Pase reaction buffer containing 3.6 mM lead nitrate, 40 mM Tris-maleate buffer, pH 6.5, 10 mM G6P, and 300 mM sucrose. After washing with 300 mM sucrose solution, the sections were colored by incubation for 1 min in 50% ammonium sulfide solution. After washing with 300 mM sucrose solution, the sections were enclosed using H1400 (Vector Laboratories, Inc) and imaged. The images of stained sections described above were captured by NanoZoomer2.0-HT (Hamamatsu Photonics). Glutaraldehyde-fixed tissues were sliced and embedded for TEM analysis following the conventional Luft embedding protocol with Quetol-812 set (Nisshin EM). The TEM images were captured by H-7500 (Hitachi High-Tech).

Metabolomic analysis. Metabolomic analysis and sample preparation were outsourced to by Human Metabolome Technologies (HMT), in accordance with HMT's C-SCOPE package and sample preparation package by the following method. Fifty microliters of plasma was added to 200 µL of methanol containing internal standards (H3304-1002, HMT) at 0°C to suppress enzymatic activity. The extract solution was thoroughly mixed with 150 µL of Milli-Q water, after which 300 µL of the mixture was centrifugally filtered through a Millipore 5-kDa cut-off filter (ULTRAFREE MC PLHCC, HMT) at 9,100*g* and 4ºC for 120 min to remove macromolecules. Besides, weighed frozen tissue (20–40 mg) was placed in a homogenization tube, along with zirconia beads. Next, 45 µL of 50% acetonitrile/Milli-Q water containing internal standards (H3304-1002, HMT) to 1 mg of tissue was added to the tube, after which the tissue was completely homogenized at 1,500 rpm and 4ºC for 60 s using Shake Master NEO (Bio Medical Science). The homogenate was then centrifuged at 2,300*g* and 4ºC for 5 min. Subsequently, 800 µL of upper aqueous layer was centrifugally filtered through a Millipore 5-kDa cut-off filter (UltrafreeMC-PLHCC, HMT) at 9,100*g* and 4ºC for 180 min to remove

macromolecules. The filtrate from plasma or tissue was evaporated to dryness under a vacuum and reconstituted in 50 μL of Milli-Q water for metabolomic analysis

Metabolomic analysis was conducted in accordance with HMT's C-SCOPE package, using capillary electrophoresis time-of-flight mass spectrometry (CE-TOFMS) for cation analysis and CE-tandem mass spectrometry (CE-MS/MS) for anion analysis based on the methods described previously (49, 50). Briefly, CE-TOFMS and CE-MS/MS analyses were carried out using an Agilent CE capillary electrophoresis system equipped with an Agilent 6210 time-of-flight mass spectrometer (Agilent Technologies) and Agilent 6460 Triple Quadrupole LC/MS (Agilent Technologies), respectively. The systems were controlled by Agilent G2201AA ChemStation software version B.03.01 for CE (Agilent Technologies) and connected by a fused silica capillary (50 μm i.d. × 80 cm total length) with commercial electrophoresis buffer (H3301-1001 and I3302-1023 for cation and anion analyses, respectively, HMT) as the electrolyte. The time-of-flight mass spectrometer scanned from m/z 50 to 1,000 and the triple quadrupole mass spectrometer was used to detect compounds in dynamic MRM mode. Peaks were extracted using MasterHands, automatic integration software (Keio University, Tsuruoka, Japan) (51), and MassHunter Quantitative Analysis B.04.00 (Agilent Technologies) in order to obtain peak information including m/z, peak area, and migration time (MT). Signal peaks were annotated in accordance with HMT's metabolite database based on their m/z values and MTs. The peak area of each metabolite was normalized to internal standards, and metabolite concentration was evaluated by standard curves with three-point calibrations using each standard compound. Hierarchical cluster analysis (HCA) and principal component analysis (PCA) (52) were performed by HMT's proprietary MATLAB and R programs, respectively.

The abbreviation of each metabolite is as follows: Pi: inorganic phosphate, UDP-Glc: uridine diphosphate-glucose, G1P: glucose 1-phosphate, G6P: glucose 6-phosphate, Ru5P ribulose 5-phosphate, F6P: fructose 6-phosphate, X5P: xylulose 5-phosphate, R5P: ribose 5-phosphate, PRPP:

phosphoribosyl 1-disphosphate, F1,6P: fructose 1,6-diphosphate, DHAP: dihydroxyacetone phosphate, GAP: glycolaldehyde 3-phosphate, 3PG: glycerate 3-phosphate, AcCoA: acetyl CoA, PEP: phosphoenolpyruvate.

Measurement of DS-4108b concentration by hybridization-based ligand binding assay and PK analysis. Frozen tissue was weighed and homogenized with beads and Buffer RLT Plus (QIAGEN) at 10 µL/mg tissue and 1,100 rpm for 60 s using Shake Master NEO. The homogenate was diluted 10-fold with Buffer RLT Plus and then centrifuged (21,600*g*, 2 min, 4°C). Five microliters of the supernatant or 5 µL of plasma was mixed with 5 µL of the complementary probe (500 nM, GeneDesign) to DS-4108b and 40 µL of HEPES Buffer (pH 7.4) and incubated (95°C for 5 min, 42°C for 60 min). Then, 10 µL of 240 U/µL S1 Nuclease (Promega) was added and incubated at 37 °C for 60 min), followed by centrifugation at 1,200*g* for 10 min at 4°C. A mixture of 5 µL of the supernatant from centrifugation and 5 µL of Rexxip F (Gyros Protein Technologies) was subjected to measurement using Gyrolab xP workstation (Gyros Protein Technologies) in accordance with the instrument protocol (200-1W-002-A). Samples exceeding the upper limit of measurement were further diluted with blank plasma or a homogenate solution prepared from untreated animals and measured again. The lower limit of quantification in each measurement was 0.3 nM in plasma or 33 pmol/g liver. For the samples with a concentration that was not detected or below the lower limit, the result was treated as zero in statistical analysis and the calculation of PK parameters. All PK parameters were calculated by Phoenix™ 64 (Build 6.3.0.395, WinNonlin 6.3, Certara).

General toxicity studies using mice and monkeys

General toxicity studies using C57B6/J mice or cynomolgus monkeys were performed at Shin Nippon Biomedical Laboratories, Ltd. DS-4108b or saline as vehicle was subcutaneously administered once every 2 weeks for 3 months to C57B6/J mice at dose levels of 10, 30, and 100 mg/kg or cynomolgus

monkeys at dose levels of 3, 10, and 30 mg/kg. A necropsy was conducted 2 weeks after the last administration. The reversibility of the toxic changes was also assessed in a subsequent 3-month recovery period in mice and monkeys. As safety pharmacology, the impact on the central nervous system was evaluated in the mice and the impacts on cardiovascular and respiratory systems were assessed in the monkeys. All items evaluated in these studies are shown in Table 1. The mice were purchased from Jackson Laboratory Japan and the monkeys were generated in Shin Nippon Biomedical Laboratories, Ltd.

In silico off-target gene candidate analysis. Sequence searches were performed using glsearch (ver. 3.6, fasta-36.3.8h, parameters: −n −3 −E 1000000000, criteria: number of mismatches/gap nucleotides ≤ 2) at Daiichi Sankyo RD Novare Co., Ltd. As a nucleotide sequence database, 160,999 (*Homo sapiens*), 76,280 (*Macaca fascicularis*), and 121,909 (*Mus musculus*) sequences were acquired from RefSeq [NOT biomol_genomic(PROP), as of 15th May, 2020]. Annotation of human genes associated with diseases or disease phenotypes in animal models was performed using RefSeq, PubMed, and MedGen.

Supplemental references

49. Ohashi Y, et al. Depiction of metabolome changes in histidine-starved Escherichia coli by CE-TOFMS. Mol Biosyst. 2008;4(2):135–147.

50. Ooga T, et al. Metabolomic anatomy of an animal model revealing homeostatic imbalances in dyslipidaemia. Mol Biosyst. 2011;7(4):1217–1223.

51. Sugimoto M, et al. Capillary electrophoresis mass spectrometry-based saliva metabolomics identified oral, breast and pancreatic cancer-specific profiles. Metabolomics. 2010;6(1):78–95.

52. Yamamoto H, et al. Statistical hypothesis testing of factor loading in principal component analysis and its application to metabolite set enrichment analysis. BMC Bioinformatics. 2014;15:51.

Supplemental Figures

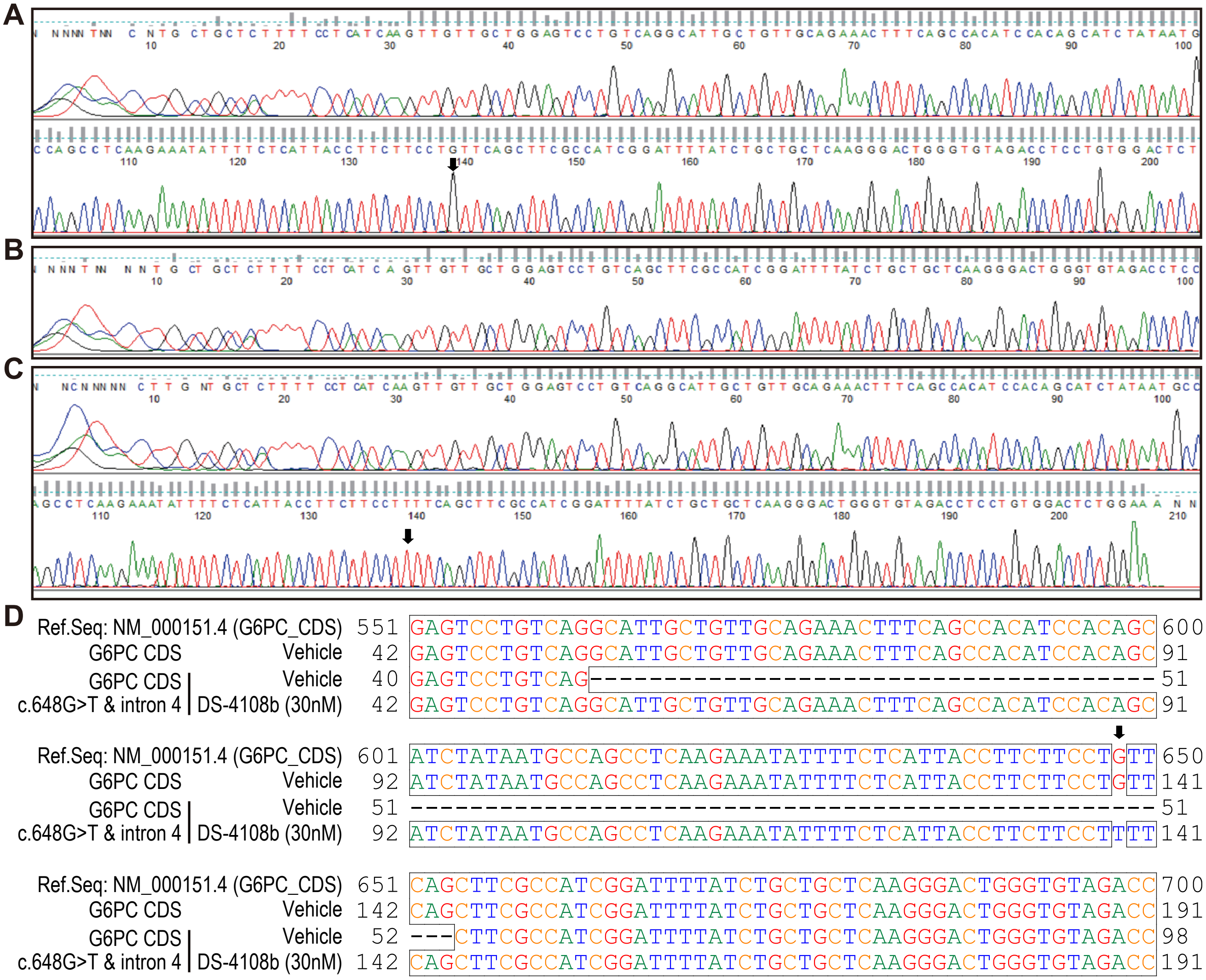

Supplemental Figure 1. Sequence analysis of RT-PCR amplicon of correctly or aberrantly spliced *G6PC* mRNA. (**A–C**) Waveform data in sequence analysis of the extracted RT-PCR amplicons of *G6PC* mRNA from agarose gel. Waveform data from the band around 250 bp in the lane of *G6PC* CDS-transfected cell lysate, the band around 150 bp in the lane of *G6PC* containing c.648G>T and intron 4 plasmid and vehicle-transfected cell lysate, and the band around 250 bp in *G6PC* containing c.648G>T and intron 4 plasmid and 30 nM DS-4108b-transfected cell lysate are shown in (**A**), (**B**), and (**C**), respectively. (**D**) Alignment analysis of the sequence data from the RT-PCR amplicons with the reference sequence (NM_000151.4). Black arrows in (**A**), (**B**), and (**D**) indicate the 648th nucleotide from the beginning of the coding sequence.

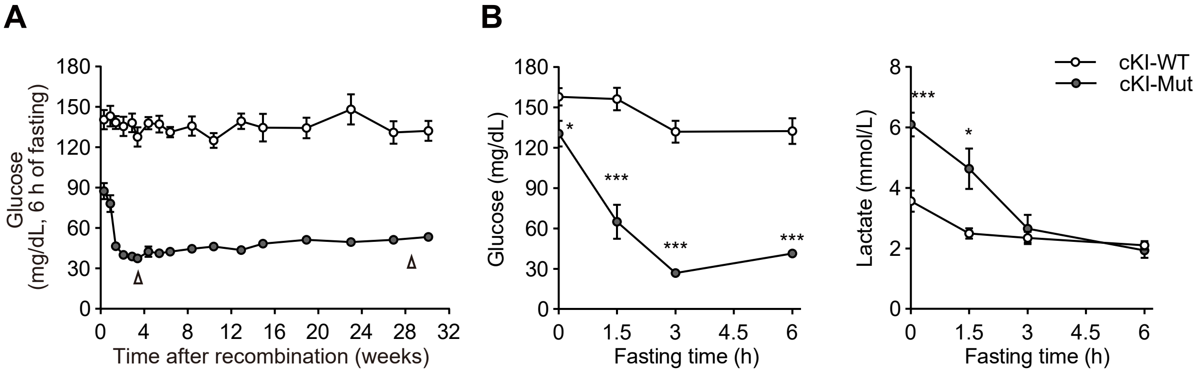

Supplemental Figure 2. Fasting blood glucose and lactate in cKI-Mut mice. (**A**) Time course changes after recombination treatment of blood glucose levels under 6 h of fasting condition of cKI-WT (*n* = 8) and cKI-Mut (*n* = 4–6) mice. White triangles represent sudden death of cKI-Mut mice. Data are presented as mean ± SEM. (**B**) Blood glucose and lactate levels of cKI-WT and cKI-Mut mice in 6 h of fasting test 2 weeks after the recombination treatment. Data are presented as mean ± SEM (*n* = 6) and were analyzed by unpaired two-tailed *t*-test to compare cKI-WT and cKI-Mut mice. Statistically significant differences with $P < 0.05$ and $P < 0.001$ are represented by * and ***, respectively.

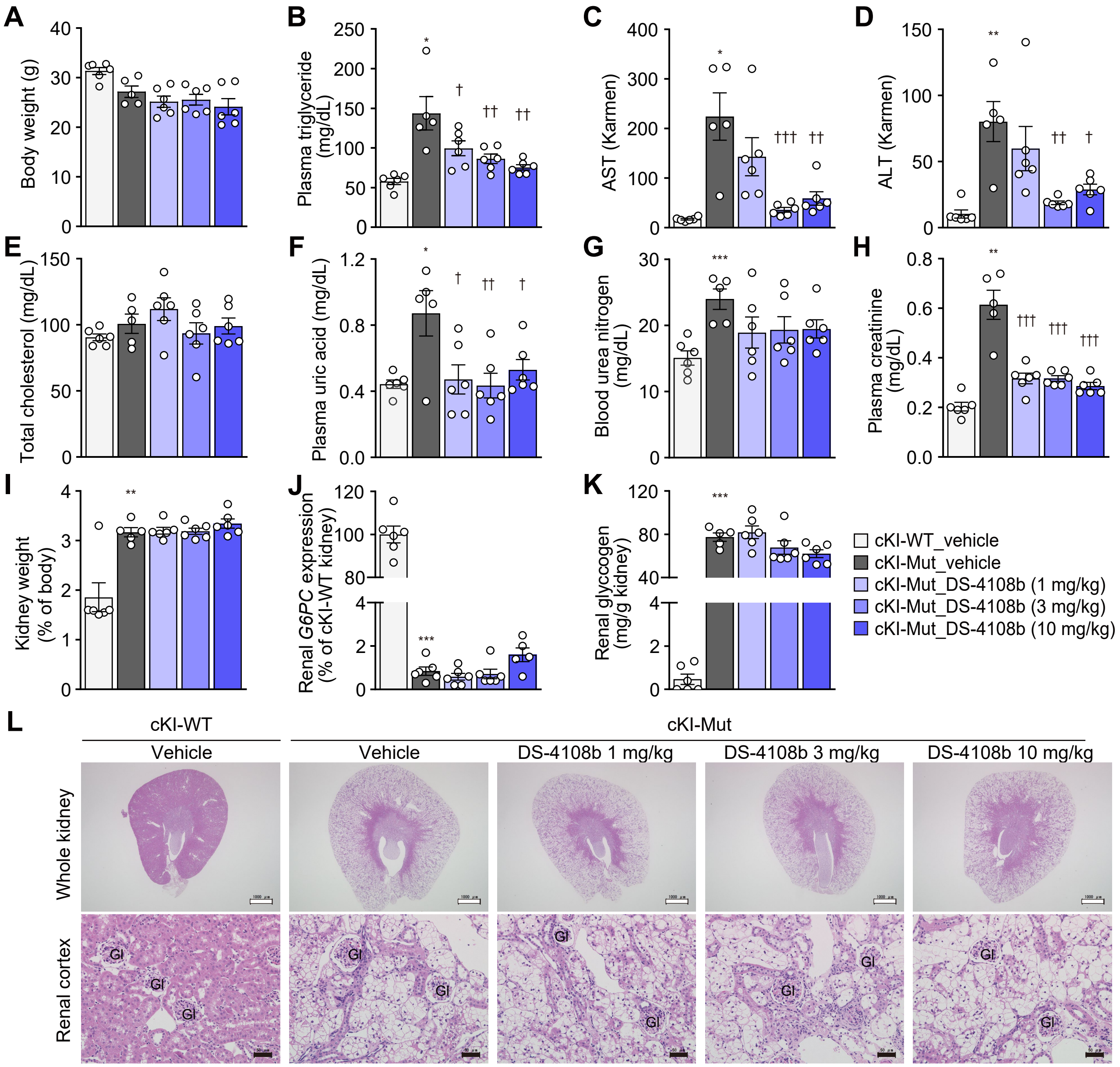

Supplemental Figure 3. Therapeutic effect of multiple doses of DS-4108b on cKI-Mut mice. (**A**) Body weight. (**B–H**) Plasma concentrations of triglyceride (**B**), aspartate aminotransferase (AST) (**C**), alanine aminotransferase (ALT) (**D**), total cholesterol (**E**), uric acid (**F**), blood urea nitrogen (**G**), and creatinine (**H**). (**I**) Weight of both kidneys relative to body weight. (**J**) Correctly spliced human *G6PC* gene expression in kidney relative to the mean of cKI-WT mice. (**K**) Renal glycogen concentration. All quantification results are presented as mean ± SEM (n = 5–6). Vehicle-treated cKI-Mut mouse group was compared with vehicle-treated cKI-WT mouse group by unpaired two-tailed t-test. Statistically significant differences with $P < 0.05$, $P < 0.01$, and $P < 0.001$ are represented by *, **, and ***, respectively. DS-4108b-treated cKI-Mut mouse groups were compared with vehicle-treated cKI-Mut mouse group by Dunnett's multiple comparison test. Statistically significant differences with $P < 0.05$, $P < 0.01$, and $P < 0.001$ are represented by †, ††, and †††, respectively. (**L**) Representative images of H&E-stained FFPE sections of whole kidney and renal cortex. Gl: glomerulus. White and black scale bars represent 1000 μm and 50 μm, respectively.

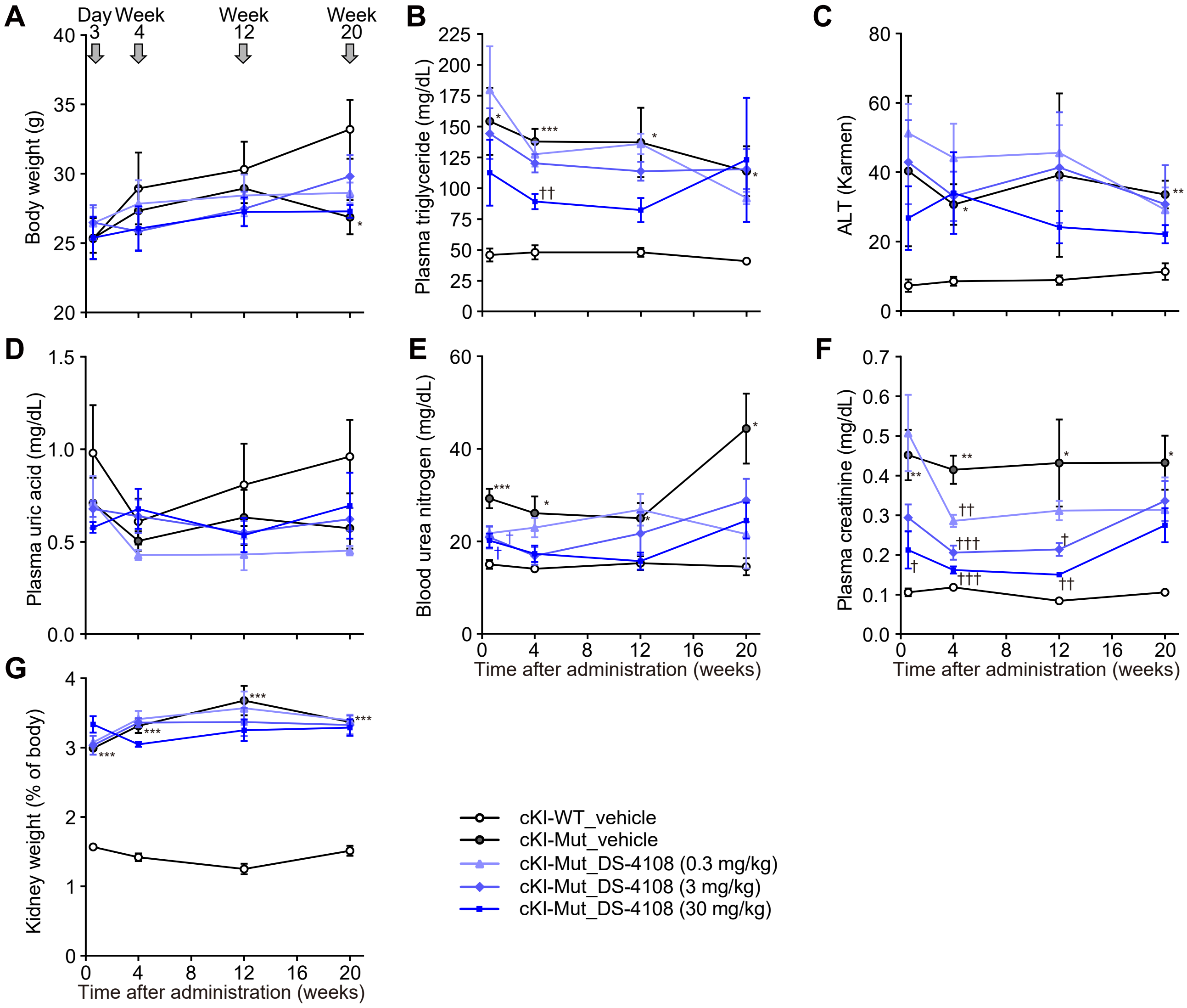

Supplemental Figure 4. Time-dependent changes of the effect of DS-4108b administered a single time on cKI-Mut mice. (**A**) Body weight and (**B**–**F**) Plasma concentrations of triglyceride (**B**), alanine aminotransferase (ALT) (**C**), uric acid (**D**), blood urea nitrogen (**E**), and creatinine (**F**) on the sampling days. (**G**) Weight of both kidneys relative to body weight of cKI-WT or cKI-Mut mice at each sampling point. For all line graph panels, quantification results are presented as mean ± SEM (n = 4–5). Vehicle-treated cKI-Mut mouse group was compared with vehicle-treated cKI-WT mouse group by unpaired two-tailed t-test at each sampling point. Statistically significant differences with $P < 0.05$, $P < 0.01$, and $P < 0.001$ are represented by *, **, and ***, respectively. DS-4108b-treated cKI-Mut mouse groups were compared with vehicle-treated cKI-Mut mouse group by Dunnett's multiple comparison test at each sampling point. Statistically significant differences with $P < 0.05$, $P < 0.01$, and $P < 0.001$ are represented by †, ††, and †††, respectively.

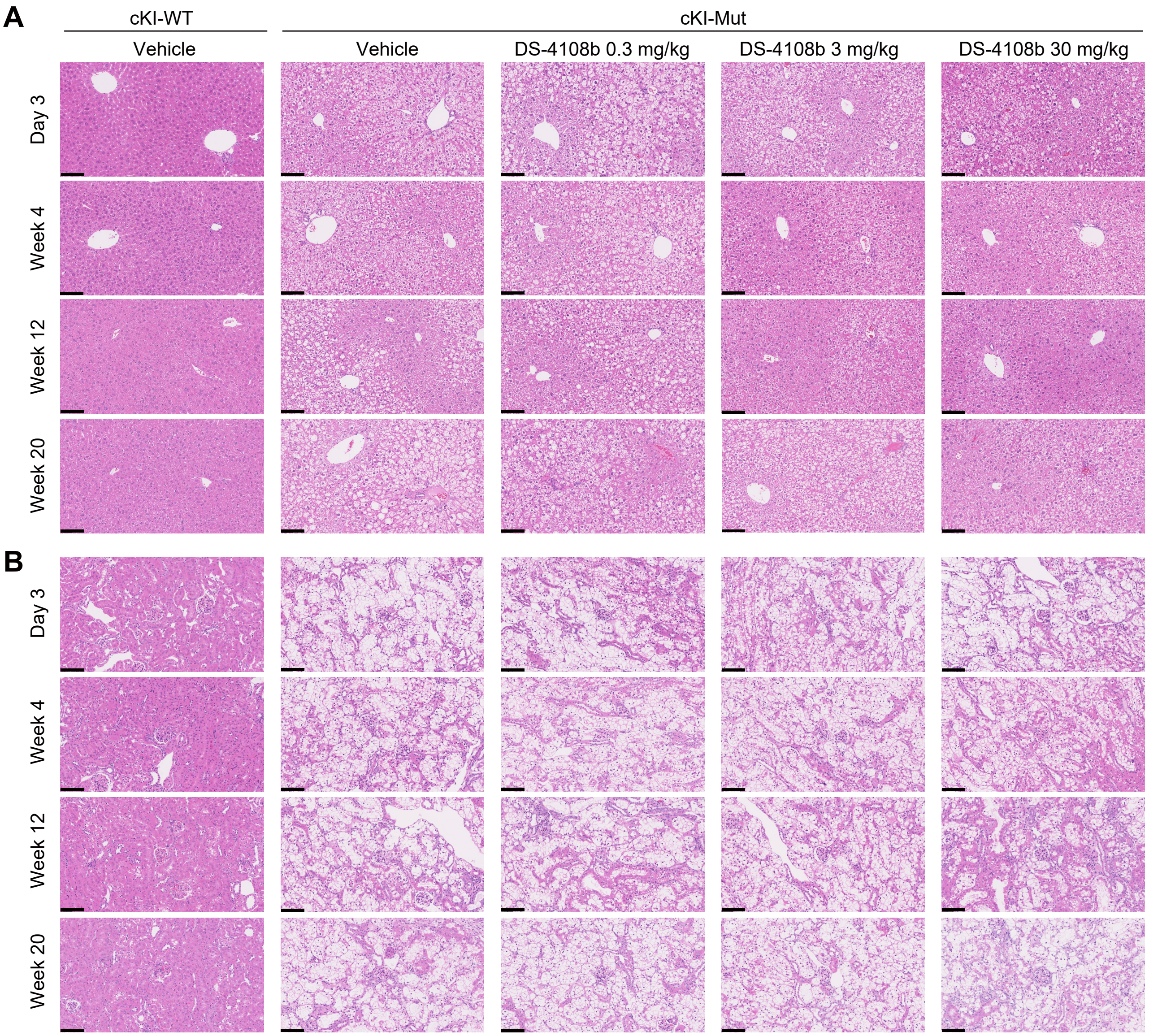

Supplemental Figure 5. Histopathology of liver and kidney of cKI-Mut mice administered DS-4108b a single time. (A, B) Representative image of H&E-stained FFPE sections of liver (**A**) and kidney (**B**) from each group at each sampling point. Black scale bars represent 100 µm.
